# PECAN Predicts Patterns of Cancer Cell Cytostatic Activity of Natural Products Using Deep Learning

**DOI:** 10.1021/acs.jnatprod.3c00879

**Published:** 2024-02-13

**Authors:** Martha Gahl, Hyun Woo Kim, Evgenia Glukhov, William H. Gerwick, Garrison W. Cottrell

**Affiliations:** † Department of Computer Science and Engineering, University of California San Diego, La Jolla, California 92093, United States; ‡ Center for Marine Biotechnology and Biomedicine, Scripps Institution of Oceanography, University of California San Diego, La Jolla, California 92093, United States; ¶ College of Pharmacy and Integrated Research Institute for Drug Development, Dongguk University-Seoul, Gyeonggi-do 04620, Republic of Korea; § Skaggs School of Pharmacy and Pharmaceutical Sciences, University of California San Diego, La Jolla, California 92093, United States

## Abstract

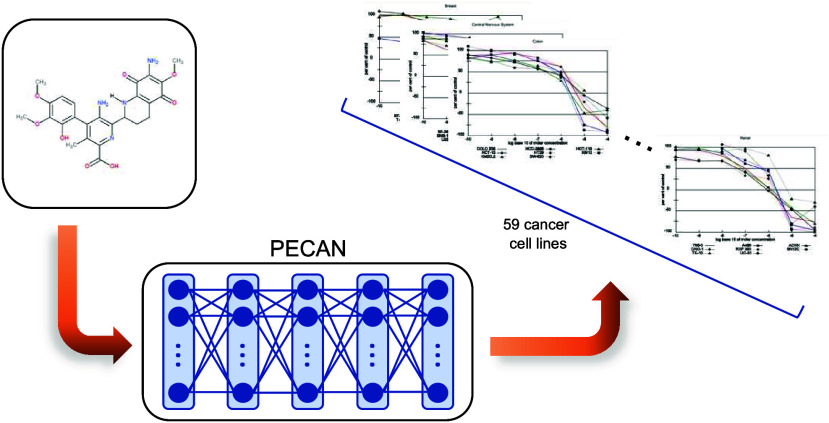

Many machine learning techniques are used as drug discovery tools with the intent to speed characterization by determining relationships between compound structure and biological function. However, particularly in anticancer drug discovery, these models often make only binary decisions about the biological activity for a narrow scope of drug targets. We present a feed-forward neural network, PECAN (Prediction Engine for the Cytostatic Activity of Natural product-like compounds), that simultaneously classifies the potential antiproliferative activity of compounds against 59 cancer cell lines. It predicts the activity to be one of six categories, indicating not only if activity is present but the degree of activity. Using an independent subset of NCI data as a test set, we show that PECAN can reach 60.1% accuracy in a six-way classification and present further evidence that it classifies based on useful structural features of compounds using a “within-one” measure that reaches 93.0% accuracy.

Cancer cells vary widely in their sensitivity to different types of chemotherapeutic agents as a result of their biochemical defects, cellular origin, and tissue location. This can make anticancer drug discovery a time-consuming and challenging process. While studies over decades have sought to characterize the biological properties of small molecules, including natural products (from bacteria, plants, animals, etc.), their derivatives, and synthetics, there are still many known compounds whose biological properties remain unknown, in addition to the many new compounds that are continually being discovered and characterized.^1^ Performing in vitro assays to evaluate the activity of new compounds to a variety of different cancer cell lines can take extensive amounts time and resources with no guarantees of the usefulness of the compound to contribute to effective cancer chemotherapy.

In order to speed and assist with antineoplastic drug discovery, we created a deep network, PECAN (Prediction Engine for the Cytostatic Activity of Natural product-like compounds), trained on the National Cancer Institute’s NCI-60 Human Tumor Cell Lines data set.^2,3^ The NCI-60 data set is an in vitro data set comprising the results from evaluation of thousands of compounds and including multiple measurements of biological activity for each cell line. PECAN uses the structure of the compound to predict the GI_50_ concentration (the concentration at which cancer cell growth is inhibited by 50%) for each of 59 different cancer cell lines. PECAN uses as inputs Morgan fingerprints, 1D vector representations of the structural components present in a compound, and performs a multiway classification to predict the activity level for each cell line. After training, PECAN can be used on unseen compounds whose antiproliferative properties have not yet been characterized to predict their activity.

The challenges that accompany traditional drug discovery methods have led many researchers to embrace computational methods as a means to speed characterization of natural products. Research using computational tools to study natural products extends past cancer research into antibiotics, antifungals, and more.^4−8^ Many of these use machine learning models to learn from existing data sets and generalize to unseen natural products and related compounds.^4,6−11^

In Walker and Clardy, logistic regression, support vector machines, and random forest classifiers were used to map biosynthetic gene clusters to several types of bioactivity. Multiple classifiers were constructed to make binary (active/inactive) predictions on antibacterial, antifungal, antitumor, and cytotoxic activity.^7^

While Walker and Clardy did not use neural networks, several other approaches to drug discovery have incorporated these into their models. Stokes et al. used a graph neural network and ensembling to use compounds’ SMILES strings and predict if they would inhibit the growth of *E. coli*.^6^ Dias et al. used a natural product data set to train two different machine learning approaches to predict the inhibitory ability of compounds against methicillin-resistant *Staphylococcus aureus* (MRSA).^4^ The first used molecular descriptors (physicochemical properties of the molecules) for a quantitative structure–activity relationship (QSAR) regression model that predicted minimum inhibitory concentration. The second used NMR descriptors and averaged the predictions of three machine learning models [random forest, support vector machine, and convolutional neural network (CNN)] to create a classification model to make binary predictions about their compounds’ anti-MRSA activity (i.e., inactive or moderately active to active).^4^ Fernández-Llaneza et al. trained a Siamese neural network, with a bidirectional long–short-term memory LSTM model, to predict the similarity in activity between two compounds using SMILES strings as inputs.^5^ The network makes binary predictions about activity. Unlike other studies, Fernández-Llaneza et al. focused on the architecture more than a particular drug target, training and testing the same architecture on five different data sets. These data sets targeted specific molecular targets involved in Alzheimer’s disease (β-site amyloid precursor protein (AAP) cleaving enzyme 1, BACE1), inflammatory diseases (CC chemokine receptor 5, CCR5), neurological diseases (dopamine D2 receptor, DRD2), cancer (epidermal growth factor receptors, EGFR), and liver disease (nuclear receptor subfamily 1 group H member 2, NR1H2).^5^

Within cancer research, machine learning models can be used to narrow down the potential pool of active compounds or to focus a search on specific cell lines. They can also be used for feature selection. Many computationally aided anticancer drug discovery studies have used machine learning techniques, although few make use of the pattern recognition capabilities provided by neural networks.

Yue et al. used 2D chemical features of compounds as input to multiple machine learning models: decision trees, support vector machines, random forests, and rotation forests. These were used to make binary determinations about the resistivity of hundreds of cell lines to different natural products.^11^

Davis et al. used a multiple linear regression analysis model to determine the optimal molecular descriptors for QSAR models. After determining optimal descriptors, they were used with a QSAR model to predict the IC_50_ of natural products against six different cell lines. Here, each cell line was predicted using separately trained models.^10^

Similarly to Dias et al., Cruz et al. trained two QSAR models, the first using molecular descriptors and the second using NMR descriptors. Cruz et al., however, focused on predicting cytotoxic activity, IC_50_, against the HCT116 human colon carcinoma cell line. Machine learning algorithms, including k-nearest neighbors, random forests, and support vector machines, were only used in the model trained with molecular descriptors.^12^

Other approaches to anticancer drug discovery have used earlier versions of the NCI-60 data set that we use in this current study.^3,13^ Li and Huang describe CDRUG, an online tool for predicting anticancer activity of compounds, which uses the NCI-60 data set as a reference for making similarity judgements between compounds in the data set and unseen compounds. The activity of unseen compounds is predicted using the activities of the most similar compounds in the data set.^13^

Our work makes three improvements on previous approaches. First, we use a multilayer perceptron, a feedforward neural network. This allows us to take advantage of the ability of deep networks to learn structural patterns in compounds in order to predict activity and generalize to unseen compounds. Second, we categorize cytostatic activity into six levels instead of making binary predictions. This gives more information about a compound while also allowing for greater specificity about the differences in activity between different cell lines. Finally, PECAN predicts the antiproliferative activity levels for 59 cell lines simultaneously, which requires the network to learn generalizable features that are useful in making predictions for all 59 cell lines.

## Results and Discussion

### Results on Validation Data

For every compound, PECAN simultaneously makes 59 cell line predictions. We compare PECAN’s predictions to experimental data, or the true activity level determined in a laboratory setting. All performance metrics compare PECAN predictions to experimentally obtained results. We report three measures of performance for PECAN. The first is the overall accuracy, the percentage of correct predictions. These results are shown in Table 1.

**Table 1:** Accuracy Values, by Activity Level and Overall, for Both Experiments, Using Either Weighted Loss or Resampled Data, for 59 Cell Lines on Validation Data^a^

	Accuracy	
Activity level	Weighted^b^	Resampled^c^
Super Potent	45.0%/64.7%	**50.6%**/**79.9%**
Potent	20.25%/62.51%	**35.1%**/**80.3%**
Active	28.1%/ 69.9%	**41.1%**/**75.8%**
Mildly Active	**46.6%**/85.8%	43.6%/**86.6%**
Weakly Active	**57.6%**/**98.8%**	54.7%/95.9%
Inactive	**73.4%**/**96.3%**	69.7%/94.0%
Overall	**60.1%**/**93.0%**	58.0%/92.0%

a Results are presented as accuracy/within-one accuracy. Bolded numbers represent best results for a given activity level.

b Loss is weighted to inversely correspond to the representation of an activity level in the data set. Underrepresented activity levels (e.g., super potent and potent) are overweighted to ensure they are not ignored.

c Instead of weighting the loss, underrepresented activity levels are resampled multiple times in order to construct a data set where all activity levels are equally represented.

For the next two measures of performance, we specifically looked at activity level specific (e.g., “super potent”) labels and predictions. First, we looked at how often a prediction of “super potent” was correct. This is the precision of PECAN for this label. We also evaluated the recall of PECAN. This is the number of “super potent” true labels that exist in the data set that are correctly identified. We report these values and their corresponding “within one” values. For precision, “within one” indicates, out of all predictions that are “super potent”, the number with a true label of “super potent” or a true label of “potent”. For recall this indicates, out of all truly “super potent” examples, the number that were predicted to be either “super potent” or “potent”. The “within one” values are important in evaluating performance because if PECAN makes a mistake, a small mistake is preferred to a large one. For example, if an example is “super potent”, we would prefer the prediction for the compound–cell line pair to be “potent” instead of “inactive”. Both are incorrect, but if PECAN generally makes smaller mistakes, we can be more confident that it is using the structure of the compound to make the predictions as opposed to having utilized insignificant details from the training data set that allowed it to perform well but in a very narrow context. The precision and recall data for all activity levels are shown in Table 2.

**Table 2:** Precision and Recall Values by Activity Level for Both Experiments, Using Either Weighted Loss or Resampled Data, for 59 Cell Lines on Validation Data^a^

	Precision		Recall	
Activity level	Weighted	Resampled	Weighted	Resampled
Super Potent	**60.0%**/**81.5%**	41.5%/58.5%	45.0%/64.7%	**50.6%**/**79.9%**
Potent	**29.5%**/**74.0%**	22.1%/60.4%	20.3%/62.5%	**35.1%**/**80.3%**
Active	**42.2%**/**86.3%**	30.7%/79.1%	28.1%/69.9%	**41.1%**/**75.8%**
Mildly Active	**53.3%**/**88.3%**	50.7%/87.2%	**46.6%**/85.8%	43.6%/**86.6%**
Weakly Active	**51.5%**/96.5%	51.4%/**97.5%**	**57.7%**/**98.8%**	54.7/95.9%
Inactive	71.8%/93.0%	**73.7%**/**94.5%**	**73.4%**/**96.3%**	69.7%/94.0%

a Results are presented as precision/within-one precision or recall/within-one recall. Bolded numbers represent best results for a given activity level.

It can be argued that recall is the most important measure of performance when choosing a model for drug discovery. This is because it provides better assurance that the most potent and super potent compounds will be identified. We reasoned that obtaining a few false positives was better than missing potentially significant compounds. We used the model with the best recall for activity levels of “active” through “super potent”, which was the model trained with resampling, to test further. We tested this model on two data sets, one with a set of unseen examples from the National Cancer Institute’s NCI-60 data set (the “test set”) and the other taken from the TimTec Library, Natural Product Library-720 (NPL-720). Only the results from the NCI-60 test set are discussed in the main body of the paper, whereas all results from the NPL-720 test set can be found in the Supporting Information. The code for PECAN trained with resampled data and a checkpoint for testing uncharacterized compounds are available at https://github.com/marthagahl/PECAN.

### Results on NCI-60 Test Data

In reporting the accuracy comparison with experimental results, we again used an exact measure and a measure that accounts for small errors. Here we report the accuracy of PECAN in predicting the activity level and the within-one accuracy of PECAN. The within-one accuracy includes any examples in which the activity levels of the experimental results and the predicted results are offset by one activity level. The accuracy of PECAN on the test data is 59.9%, and the within-one accuracy is 92.9%.

**Figure 1:**
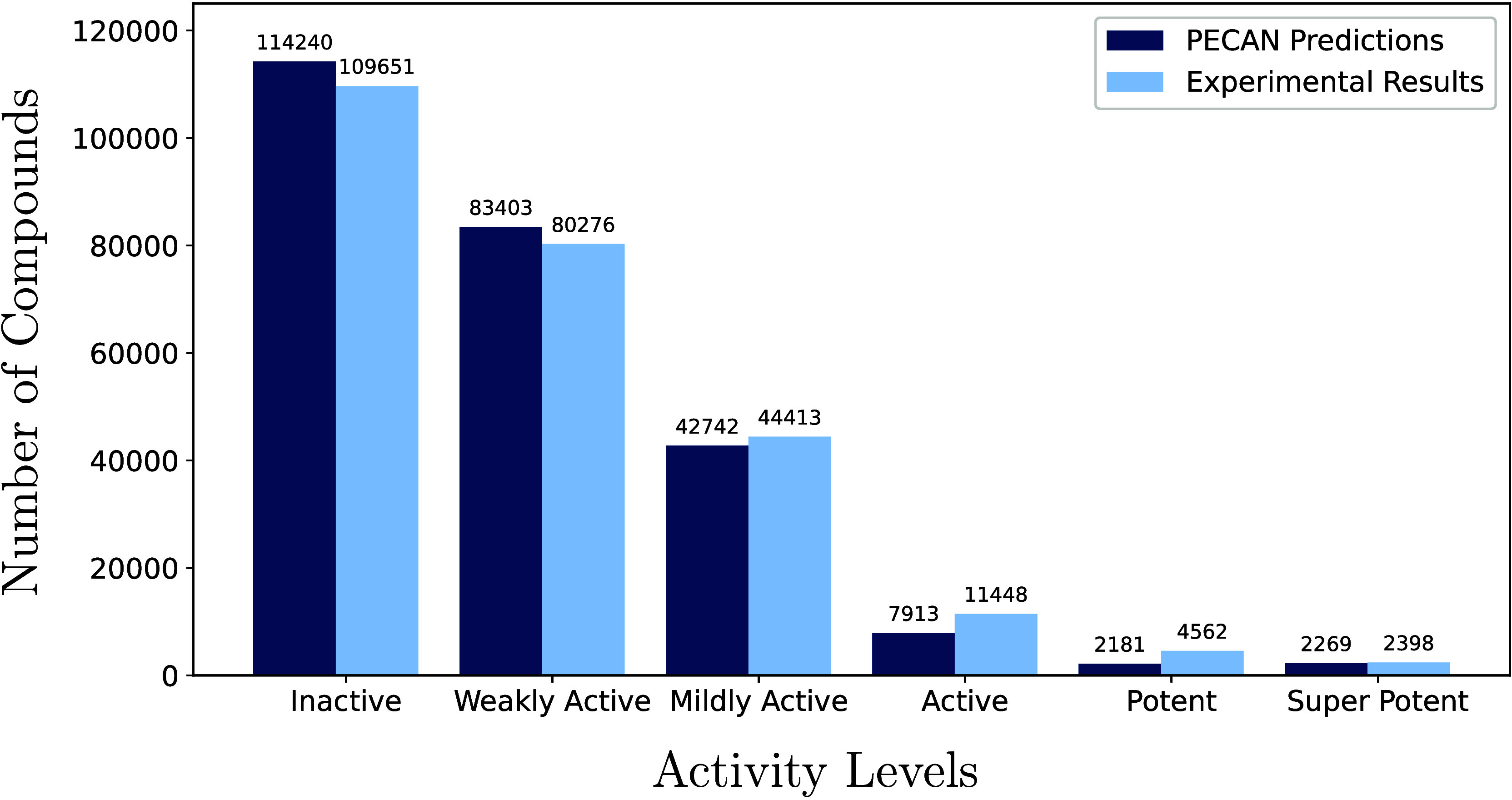
Counts of PECAN activity level predictions (averaged over cell line activities) and experimental activity levels.

**Figure 2:**
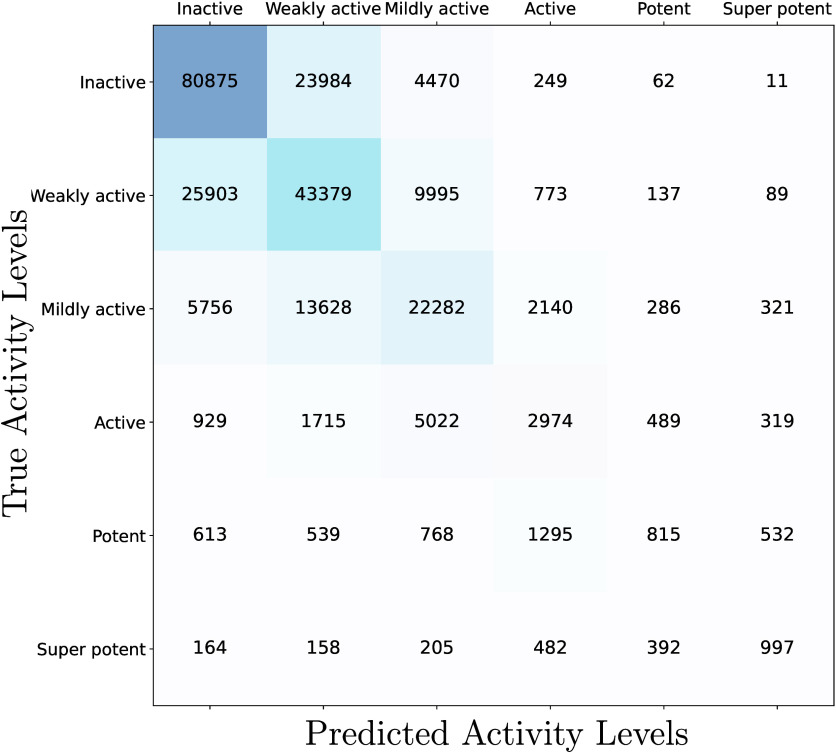
Confusion matrix for PECAN predictions using the NCI-60 test set. Columns indicate total predictions for each activity level, and rows indicate the true label for each prediction. Values on the upper left to lower right diagonal indicate correct predictions of cell line activity for a particular compound. Darkened boxes represent higher numbers of correct predictions.

Figure 1 shows the number of compounds classified into each antiproliferative activity level from PECAN predictions on the NCI-60 test set and experimental results. For PECAN predictions, in addition to recording correct predictions, we also noted the distance between incorrect predictions and their true labels. We created confusion matrices to analyze the distribution of incorrect predictions (Figure 2). In Figure 2, the columns are the predicted activity levels. These values sum to the counts shown in Figure 1. The columns indicate how many times PECAN predicted each activity level. The rows are the true labels. These values sum to the counts in the data set. For any given compound, the column indicates what its activity level was predicted to be, and the row indicates what the true activity level is. Therefore, correctly predicted compounds lie on the diagonal. Compounds that are incorrectly predicted are off-diagonal. However, the closer the incorrect predictions are to the diagonal, the closer those predictions were to the true labels.

**Figure 3:**
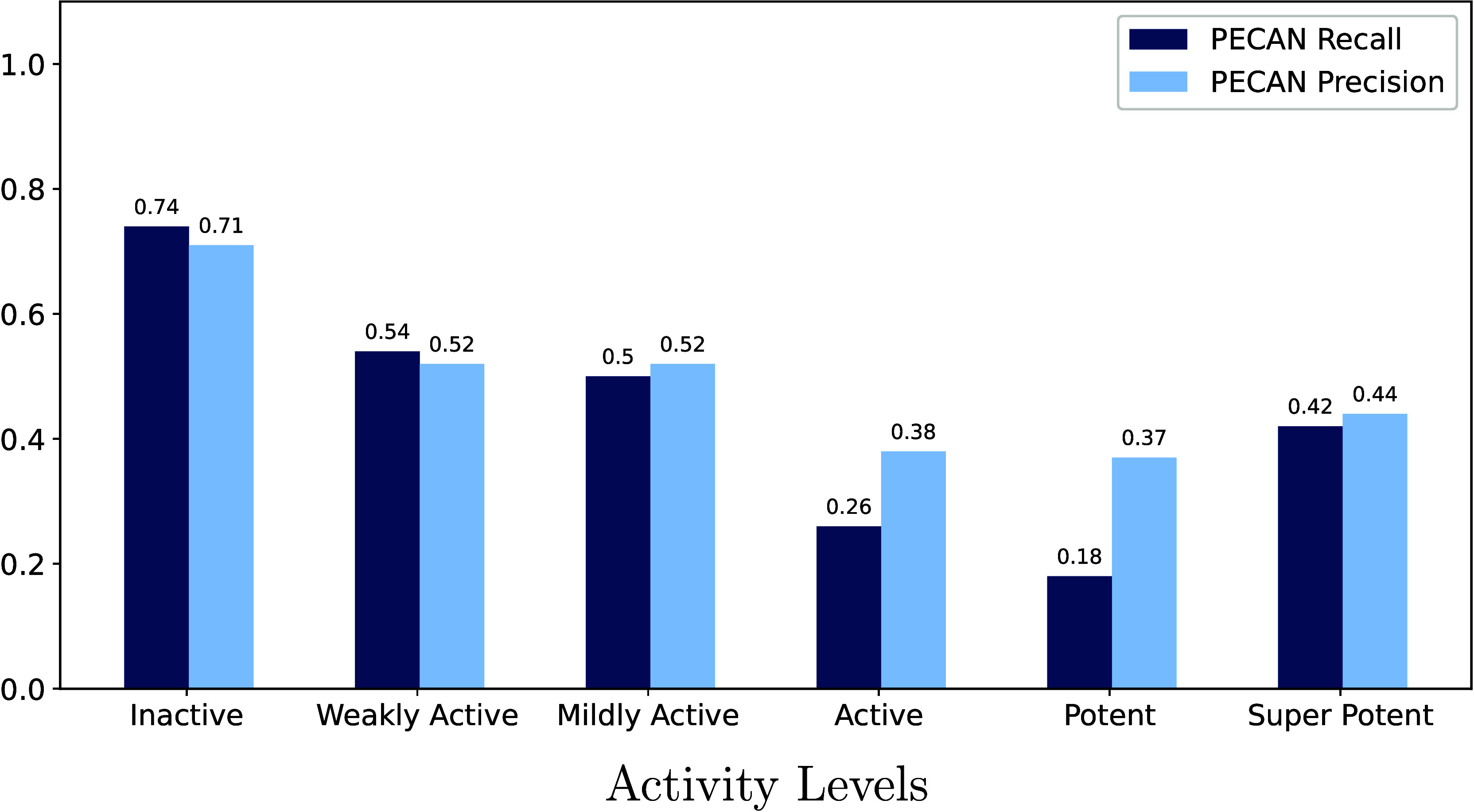
Recall and precision of PECAN predictions when compared with experimental results for the NCI-60 test set based on data shown in Figure 2.

We can more clearly analyze how well PECAN performs with precision and recall, which are plotted in Figure 3. For the test data, we report precision and recall for all activity levels.

Finally, we calculate the selectivity of the compounds in the NCI-60 test set and the selectivity of PECAN’s predictions. We use the selectivity definition of having at least one cell line GI_50_ value be lower than the average GI_50_ for the compound by two log orders. The NCI-60 test set includes 346 selective compounds by this definition out of 4913 total compounds in the test set. PECAN predicted only 5 of the 4913 compounds to be selective. We believe the lack of accuracy in PECAN’s selectivity predictions results from holes in the data and the scarcity of data for particular cell lines. More training data are needed to improve PECAN’s predictions of selective compounds.

### Discussion

We further analyzed our predictions of extreme values, inactive or super potent, and the compounds that contributed to those predictions. Each number on the confusion matrix in Figure 2 indicates an example, or a compound and cell line activity pair. While we only have fewer than 50,000 compounds total for the training, validation, and test sets, we have almost 3 million examples of compound and cell line activity pairs because each compound has 59 associated activity levels: one for each cell line. However, we can look at our confusion matrix results in terms of the compounds that make up each square. There are 164 examples that were predicted to be inactive and were truly super potent. All 164 examples come from just 52 compounds. There are 997 examples that were correctly predicted to be super potent, and all 997 of these examples came from 55 compounds. Therefore, on average each of those 55 compounds correctly predicted just over 18 super potent examples out of 59 cell lines. There are 11 examples that were predicted to be super potent and were truly inactive. These come from 6 different compounds.

Of those compounds predicted to be inactive but had true labels as super potent, there were a diversity of small heterocyclic alkaloids that appear mostly to be synthetic in origin. The compounds predicted to be super potent with true labels as super potent included such well-known cytostatic natural products as alkaloidal steroid dimers, digitoxin analogs, mithramycin-type compounds, camptothecin analogs, didemnin B analogs, cryptophycin A, colchicine analogs, epothilone analogs, taxanes, anthramycin analogs, steroidal glycosides, and actinomycin D analogs. Finally, the few compounds that were predicted to be super potent but had true labels as inactive included a tetrahydrofolate derivative, taxane derivatives, and colchicinoid-like compounds. The compound NSC IDs and structures can be found in Supporting Information Tables S1, S2, and S3.

These results are promising in their ability to narrow down the scope of compounds to test experimentally for potential evaluation as cancer therapeutics. They are also encouraging more broadly for the use of machine learning to aid in drug discovery efforts. PECAN is able to predict antiproliferative activity in multiple cancer cell lines with high recall. While there will certainly be false positives, this tool can be used to determine which compounds should be explored further and reduce the incidence of potent compounds being incorrectly excluded. The ability to screen compounds and remove those with low cytostatic activities will speed the drug discovery process by focusing time and resources on those compounds with a greater likelihood of being effective as cancer therapeutics. In addition, PECAN can be used to screen for cell line specificity. By predicting activity levels for all cell lines, PECAN gives a more comprehensive view of likely interactions with various tissues in the body. These results could allow identification of compounds with high specific activity in one or a few cell lines versus those compounds that are broadly potent and might be too toxic for use as unmodified agents. However, as noted above, the current version of PECAN does not perform well in detecting selective compounds, presumably due to incomplete and insufficient data sets. On the other hand, broadly potent compounds could be evaluated for their utility as “warheads” in antibody–drug conjugates (ADCs).

## Conclusion

By harnessing PECAN’s ability for pattern recognition, we have developed a method by which to map the structure of a compound to its potential utility to inhibit the growth of cancer cells. The same principle of mapping structure to function can be applied to other active areas of drug discovery, if sufficient training data are available. By building a model that predicts activity to multiple cell types simultaneously, we have shown that deep networks have the ability to provide a broad picture about drug interactions in different systems. This has been achieved by designing PECAN with an output that allows researchers to screen for particular activity levels as well as for activity to particular cell lines. Interesting insights are gained into potential correlations between agents and their predicted activities to specific cell lines. PECAN provides further insight into how machine learning can be widely useful to scientific questions of varying foci and goals.

## Experimental Section

### PECAN Model

PECAN uses Morgan fingerprints, 1D bit vector representations of molecules, as input. Each Morgan fingerprint is 6144 bits long. PECAN outputs predictions of activity level for 59 cell lines for each Morgan fingerprint. The predicted activity levels are vectors of length 354 because there are 59 cell lines and 6 outputs for each (i.e., 6 different levels of antiproliferative activity), which are arranged in a softmax between the six categories. This allowed a simultaneous six-way classification for the 59 different cell lines and enabled PECAN to use feedback from all 59 cell lines to learn an internal representation of the structure function relationship of each compound.

In order to determine the optimal architecture for predicting cytostatic activity, we searched over model types, architectures, and hyperparameters. We tried three types of models: regression models, multilayer perceptrons, and 1D convolutional models. We also tested having separate prediction heads with unique weights for each of the cell lines. Multiplayer perceptrons with a single output had the most robust performance and minimized computation time. We searched over architectures by considering the type of layers, the number of hidden layers, and the number of hidden units in each layer. For hyperparameters, we searched over different values of input layer dropout, hidden layer dropout, and L2 weight decay. We used the validation data to determine the optimal architecture for PECAN: a multilayer perceptron with five layers, each with 256 hidden units, using ReLU nonlinearity. It also used an input dropout of 30%, a hidden layer dropout of 60% in each layer, and a weight decay of 0.0001. This is the final architecture of PECAN.

### Data

**Figure 4:**
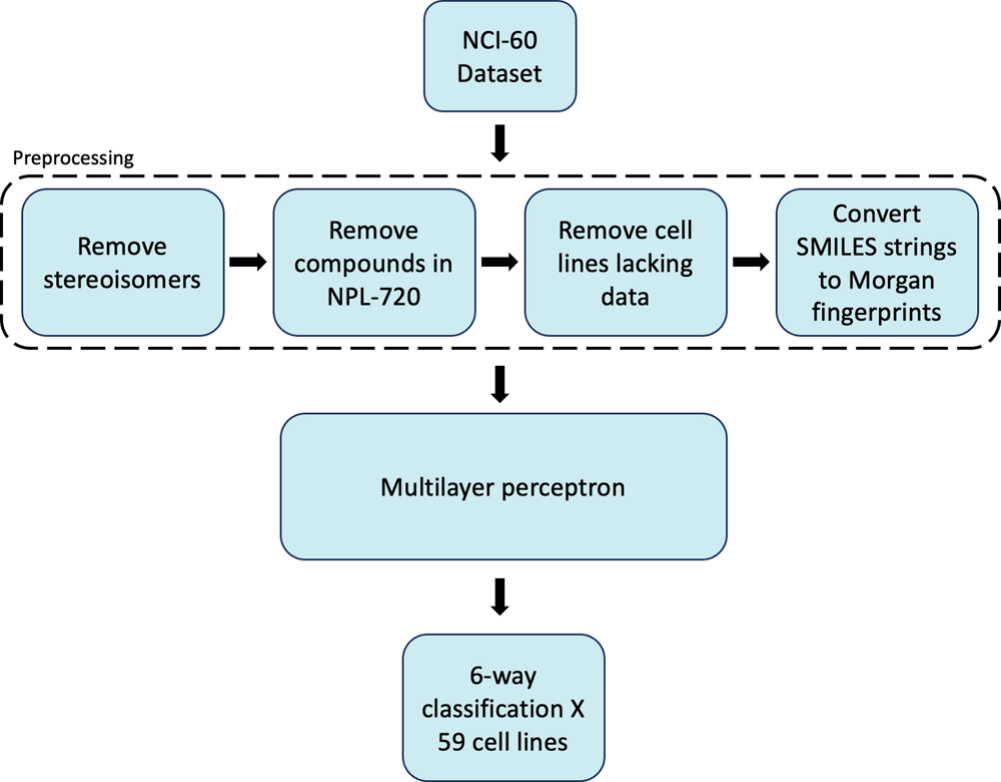
Pipeline used to preprocess data and perform experiments.

The data used in our experiments came from the National Cancer Institute’s NCI-60 data set.^3^ This data set included the NSC IDs for different small molecules, including natural products (from bacteria, plants, animals, etc.), their derivatives, and synthetics as well as the antiproliferative data for each compound for each of the cell lines in the data set. In our experiments, we used only 59 cell lines as we omitted any cell lines with fewer than 30,000 data points. The cell lines used can be found in the Supporting Information in Tables S2 and S3. We preprocessed compound IDs to remove stereoisomers as well as any compounds that were also in the TimTec Natural Product Library-720 (NPL-720).^14^ The preprocessed NCI-60 data set was randomly split into a training set, a validation set, and a test set. NPL-720 was used as an additional test set for several reasons. First, these compounds are commercially available, and second, not all of the compounds included in NPL-720 have had their antiproliferative activity to cancer cells evaluated. Because the compounds are all purchasable, but not all have their cancer cell cytostatic activity characterized, they could subsequently be evaluated in the NCI-60 cancer cell line assay. This procedure enabled potential laboratory validation for PECAN’s performance on unseen compounds. Finally, the NPL-720 is an external data set. The test set of NCI-60 will have a similar distribution to the training set, while the NPL-720 is a separate data set and should have more variation in its data. However, the NPL-720 is a small data set with very few examples of certain activity categories. The test set of NCI-60 is much larger and provides prediction results across many more compounds, leading to a more realistic demonstration of PECAN’s performance. The data preprocessing and experimental pipeline are detailed in Figure 4, and the NPL-720 results are included in the Supporting Information.

We used the SMILES strings for each of the 49,126 remaining NSC IDs in order to obtain chemical structures for all compounds. The SMILES strings were then converted to a version of Morgan fingerprints, using RDKit,^15^ which were then used as the input to PECAN. Morgan fingerprints are bit vectors created using a hashing algorithm that maps substructures in the compound to locations in the vector. A ‘0’ indicates absence of the substructure, and a ‘1’ indicates its presence. In a minor modification of the standard Morgan fingerprint, we separated out the bits for different radii (0, 1, and 2), with 2048 bits for each to try to avoid collisions.

**Figure 5:**
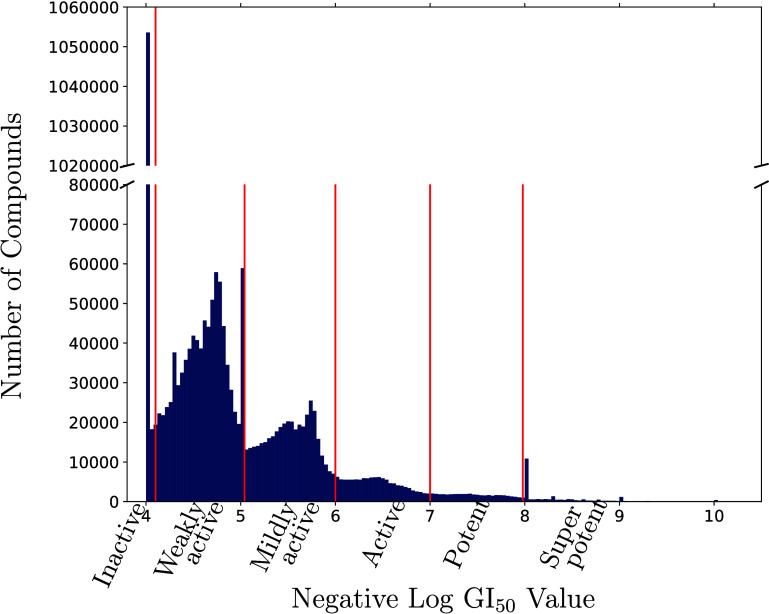
Distribution of activity levels of all compounds in the data set (blue) and the negative log GI_50_ value thresholds we used to categorize the compounds (red). Activity level annotations are given for each portion of the distribution: “Inactive”, “Weakly active”, “Mildly active”, “Active”, “Potent”, “Super potent”.

For training PECAN, we needed a representation of the negative log GI_50_ concentrations for each compound in each of the 59 cell lines. We chose to represent these as six categories of activity: inactive, weakly active, mildly active, active, potent, and super potent. The thresholds for these categories were obtained by examining the distribution of all activity levels in the data set and setting boundaries at apparent inflection points. This distribution and the selected thresholds are shown in Figure 5.

**Figure 6:**
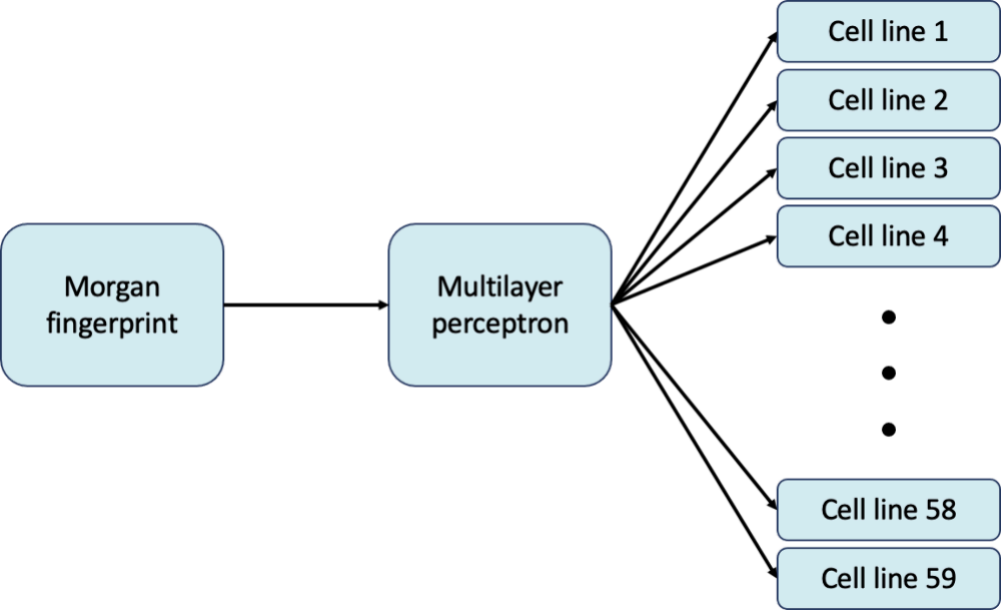
PECAN architecture for predicting the activity level for 59 cell lines. Each box labeled “Cell Line” consists of a 6-way softmax vector.

The numeric thresholds for the negative log GI_50_ concentrations are given in Table 3. These categories were then assigned to each compound based on where their activity levels fell. Hence, each compound has 59 different targets, one for each cell line. As a result, PECAN learned to predict an activity level for all 59 cell lines from one input Morgan fingerprint, as shown in Figure 6. We chose to design PECAN to predict one of six activity levels instead of making a binary determination (active or inactive) in order to provide the greatest specificity possible about the compounds’ activity levels. Training data sets with a broader range of biological values have also been shown to create better models.^16^

**Table 3:** Negative log GI_50_ Activity Category Thresholds and Dataset Activity Level Category Sizes and Percentages

Category	Negative log GI_50_ concentration	Number of examples	Percent of data set
Inactive	<4.1	1,091,610	43.21%
Weakly Active	[4.1, 5.0)	801,171	31.71%
Mildly Active	[5.0, 6.0)	450,447	17.83%
Active	[6.0, 7.0)	117,253	4.64%
Potent	[7.0, 8.0)	41,356	1.64%
Super Potent	≥8.0	24,614	0.97%

Each of the 49,126 Morgan fingerprints had 59 activity levels, one for each cell line. Where data were missing for a particular cell line, we did not provide a target for that output segment (i.e., no error was propagated back for that cell line).

The data set was extremely unbalanced, a feature that could lead to biased results in neural networks. For example, if we used the entire training set, a neural network could achieve a fairly low error by learning to categorize everything as inactive or weakly active. We used one of two methods to compensate for this bias in the data set. In the first method we resampled the minority categories (active, potent, and super potent) with replacement to provide 200,000 examples each, and we subsampled the other three categories to 200,000 examples each. As a result, the categories were perfectly balanced. We did the resampling only once and used that resampled data set for all resampling experiments. The second method used a weighting of the loss of the categories according to their frequency in the data set during training.

First, we shuffled all examples and randomly selected 80% to be in the training set, 10% to be in the validation set, and 10% to be in the test set. In instances where data were missing, or a fingerprint had not been tested with a certain cell line, we excluded this example from the loss calculation. For trials using resampling, we resampled only the training data and left the validation data and test data unbalanced.

**Figure 7:**
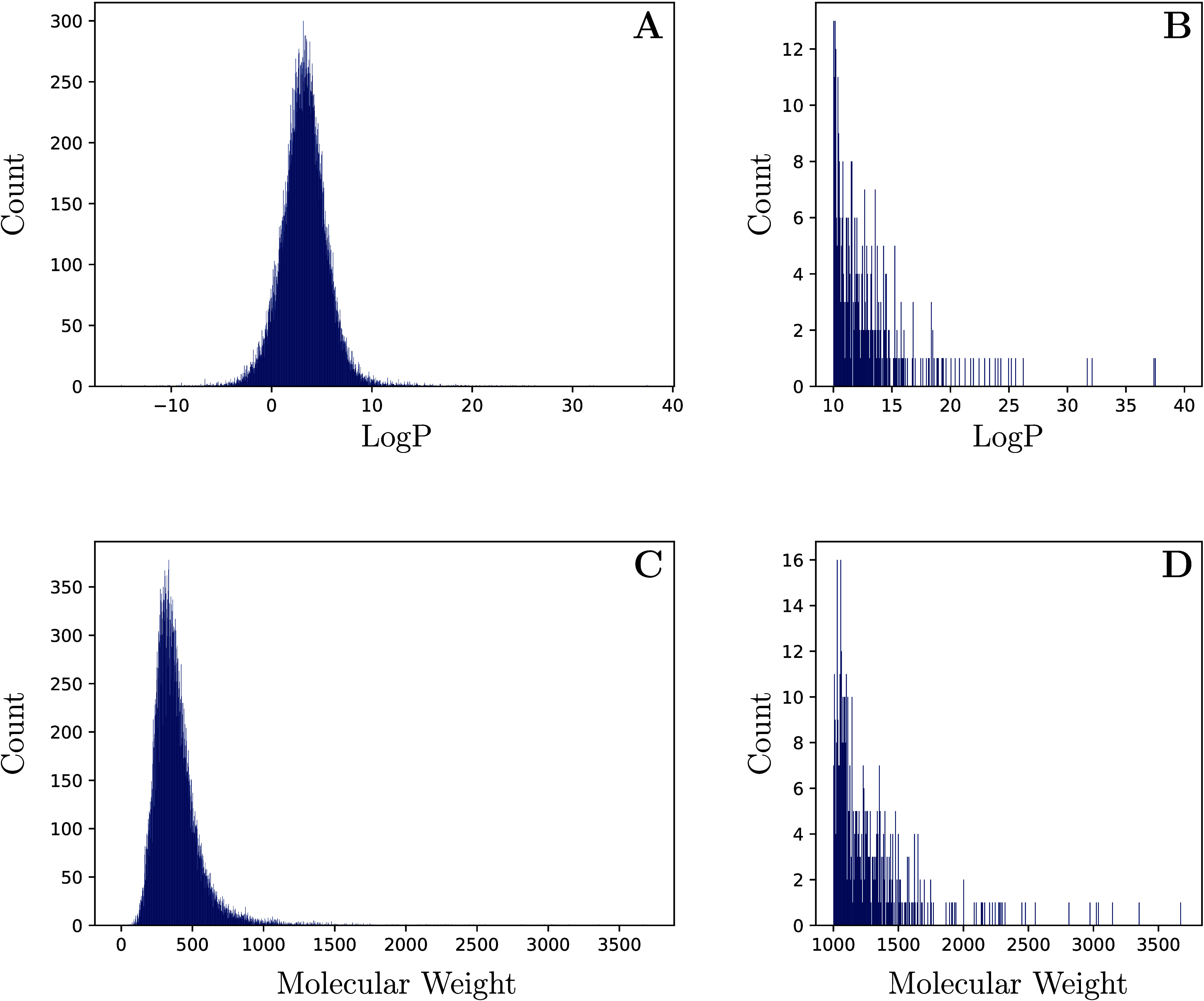
Distributions of LogP and molecular weight values in the NCI-60 data set. (A) All values of LogP. (B) Magnified view of LogP values greater than 10. (C) All values of molecular weight. (D) Magnified view of molecular weight values greater than 1000.

**Figure 8:**
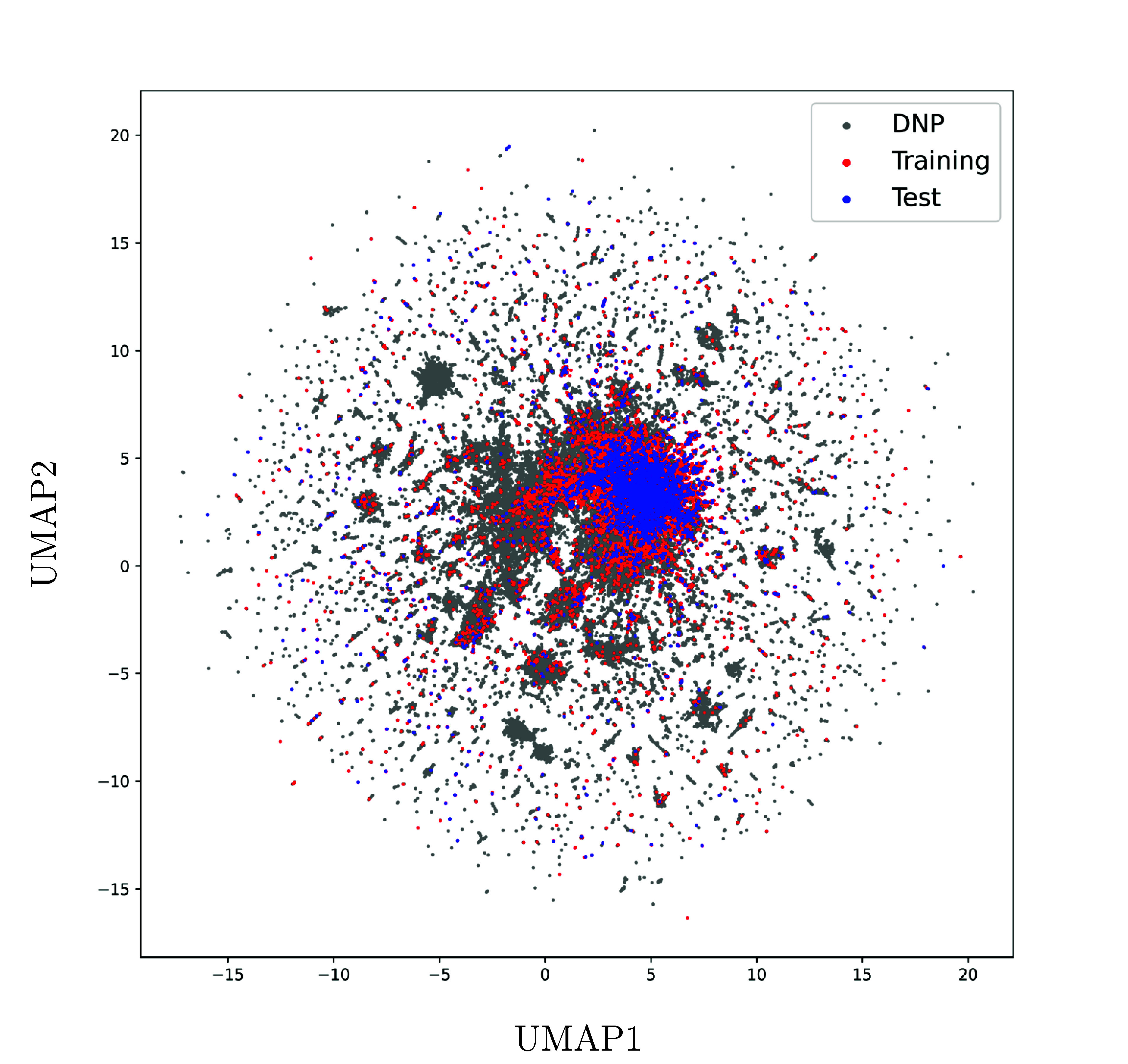
Chemical diversity for the training and test sets taken from the NCI-60 data set. Dictionary of Natural Products shown in gray, training set shown in red, and test set shown in blue. All UMAP values calculated using normal Morgan fingerprints.

To provide insight into the composition of the NCI-60 data set, we aggregate compound properties LogP and molecular weight into histograms in Figure 7. Figure 7A and C provide the distribution of LogP and molecular weight values respectively in the data set (all compounds in training, validation, and test sets). Figure 7B and D demonstrate the diversity of values represented in the data set by magnifying the extremes of the distributions. LogP and molecular weight values for the NPL-720 external test set are provided in Table S1 in the Supporting Information. We also compare the chemical diversity and distributions of the training and the test sets used in Figure 8. Here, UMAP1 and UMAP2 represent dimensions with large amounts of variation in the data set. The chemical diversity of the Dictionary of Natural Products (DNP) is also shown in gray in Figure 8. The DNP contains the majority of reported natural products, giving a broad estimation of the distribution of chemical diversity at large. Both the training and test sets show similar clustering to DNP and include examples across the entirety of the DNP distribution, suggesting the training and test sets are representative samples of reported natural products.

### Training

We performed two experiments with PECAN: one training on the original data with weighted loss and one training on resampled training data with no weighted loss. Both experiments used the same architecture and data thresholds. We used early stopping to determine the number of epochs to train and chose the Adam optimizer with an initial learning rate of 1e-5.

## Dedication

Dedicated to Professor Vanderlan da Silva Bolzani, São Paulo State University (UNESP), for her pioneering work on bioactive natural products.
