# Supplementary material for: PECAN Predicts Patterns of Cancer Cell Cytostatic Activity of Natural Products Using Deep Learning
Source: J Nat Prod. 2024 Feb 13;87(3):567–75. doi: 10.1021/acs.jnatprod.3c00879 (PMC10960629; doi:10.1021/acs.jnatprod.3c00879)
Supplement: Supplementary file 1 — np3c00879_si_001.pdf [file np3c00879_si_001.pdf]

Supporting Information
PECAN Predicts Patterns of Cancer Cell Cytostatic Activity of Natural Products using Deep Learning

Martha Gahl,[*,†] Hyunwoo Kim,[‡,¶] Evgenia Glukhov,[‡] William H. Gerwick,[‡,§] and Garrison W. Cottrell[†]

†*Department of Computer Science and Engineering, University of California San Diego, La Jolla, CA, USA*

‡*Center for Marine Biotechnology and Biomedicine, Scripps Institution of Oceanography, University of California San Diego, La Jolla, CA, USA*

¶*College of Pharmacy and Integrated Research Institute for Drug Development, Dongguk University-Seoul, Gyeonggi-do, Republic of Korea*

§*Skaggs School of Pharmacy and Pharmaceutical Sciences, University of California San Diego, La Jolla, CA, USA*

* E-mail: mgahl@ucsd.edu

List of Figures

Figure S1 PECAN NPL-720 activity level prediction distribution 20
Figure S2 PECAN NPL-720 prediction confusion matrix 20
Figure S3 PECAN NPL-720 recall and precision 21

List of Tables

Table S1 NCI-60 compounds incorrectly predicted to be inactive 10
Table S2 NCI-60 compounds correctly predicted to be super potent 17
Table S3 NCI-60 compounds incorrectly predicted to be super potent 18
Table S4 NPL-720 compounds 45
Table S5 PECAN predictions on cell lines 1-30 52
Table S6 PECAN predictions on cell lines 31-59 58

List of Supplementary Methods

Method S1 Extreme Predictions on NCI-60 Test Set 3
Method S2 Independent Test Set 3
Method S3 NPL-720 Test Set Results 19
Method S4 NPL-720 PECAN Predictions 46

Method S1. Extreme Predictions on NCI-60 Test Set: Correct and incorrect predictions at the extremes of activity levels

In the Results and Discussion section of the main paper we discuss the most extreme predictions of PECAN (inactive or super potent) and give examples of the types of compounds that included correct predictions or severely incorrect predictions. Here we present the NSC IDs of these compounds and their structures. Any additional information about the compounds themselves can be obtained by going to https://dtp.cancer.gov/dtpstandard/dwindex/index.jsp and entering the NSC ID. Table S1 contains compounds with compound-cell line pairs that PECAN predicted to be inactive but with true activity levels of super potent. Table S2 contains the compounds with compound-cell line pairs that were correctly predicted to be super potent. Table S3 contains compounds with compound-cell line pairs that PECAN predicted to be super potent but with true activity levels of inactive.

Method S2. Independent Test Set: TimTec Library Compounds

We used the TimTec Natural Product Library-720 (NPL-720) as an independent test set. It was purchased in 2014 and was comprised of 720 natural product compounds[1]. Using PECAN trained with resampled data, we predicted activity levels for all 720 compounds for each of the 59 cell lines, respectively. These results were then analyzed and compared with experimental data from the NCI database[2] to determine the level of agreement between PECAN's predictions and experimentally determined results. From the 720 compounds in NPL-720, we were able to analyze 106 compounds to compare to our predictions. Compounds from NPL-720 (179) were excluded if they did not have CAS numbers. An additional 434

NSC ID	Structure	NSC ID	Structure
677961	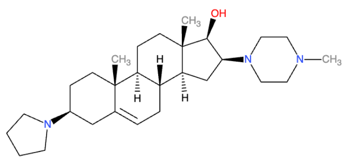	39147	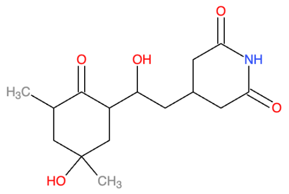
93033	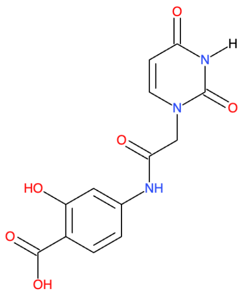	625801	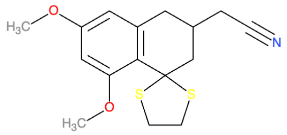
25703	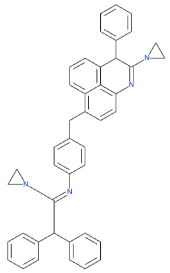	691822	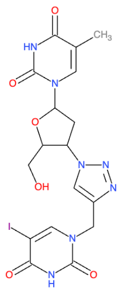
717227	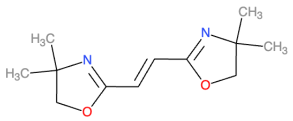	707617	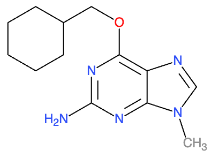

703899	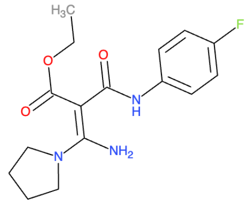	735469	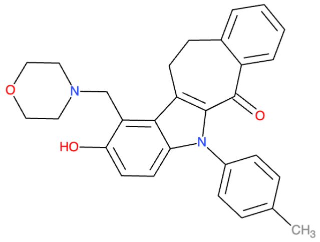
708049	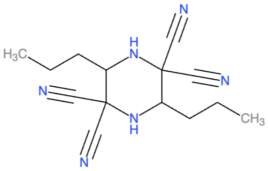	676998	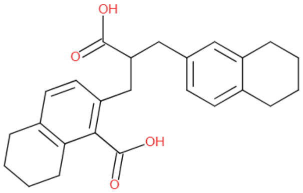
639900	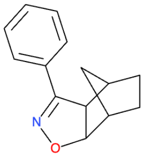	704205	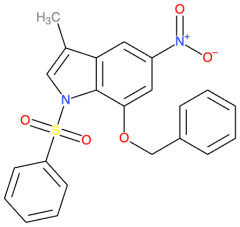
666517	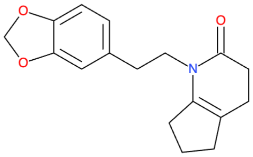	722730	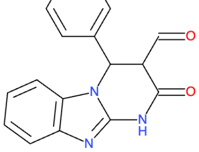

670093	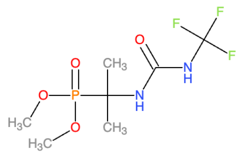	682294	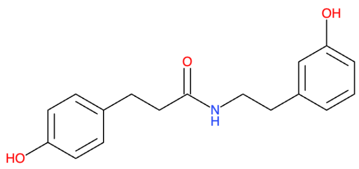
669667	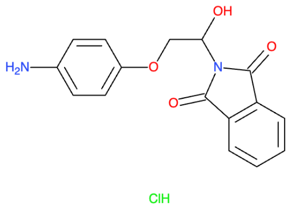	809972	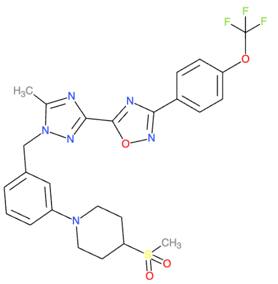
657336	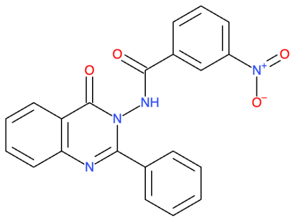	358304	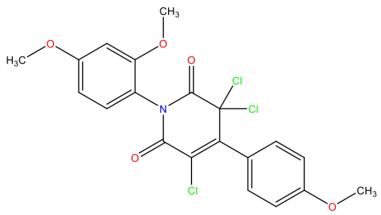
700718	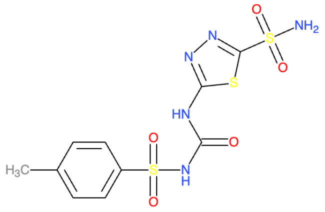	700911	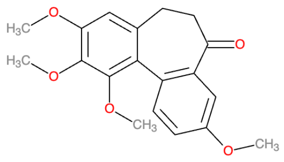

707546	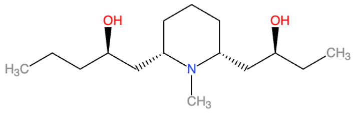	619192	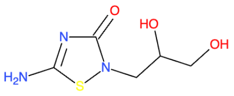
666174	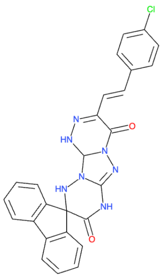	625639	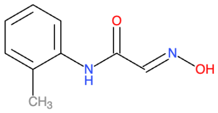
716206	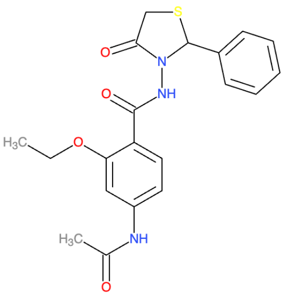	657957	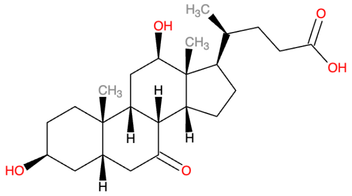
781010	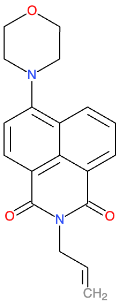	691127	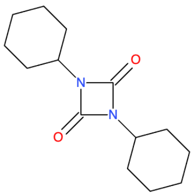

781471	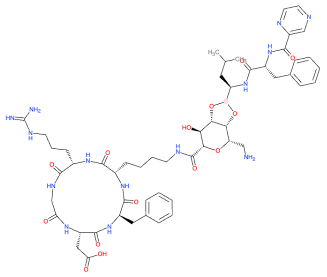	42352	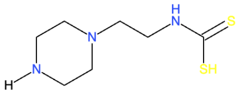
666472	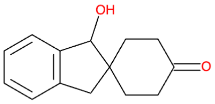	32743	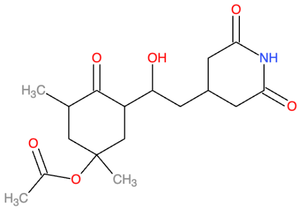
786096	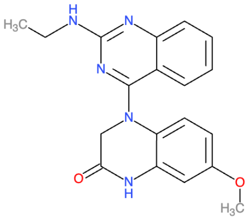	783834	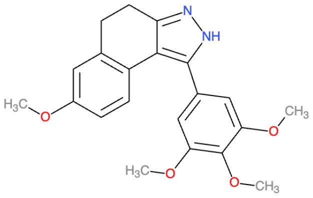
638479	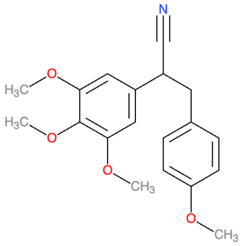	168597	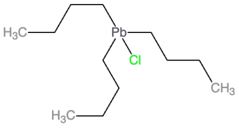

645087	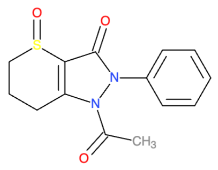	664228	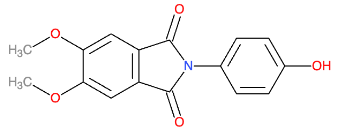
667562	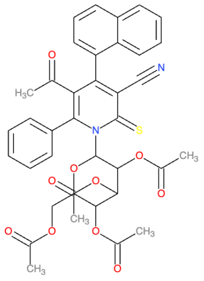	710116	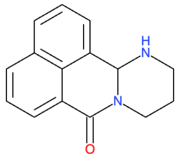
630975	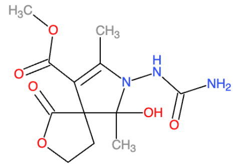	704881	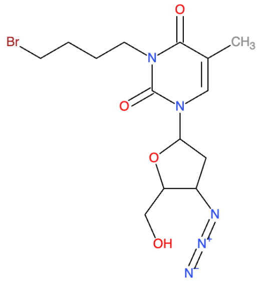
240553	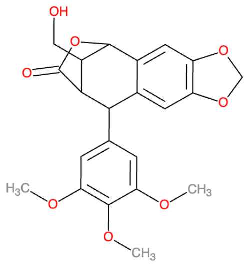	676996	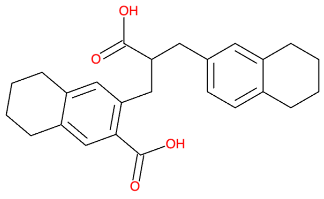

741776	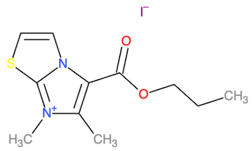	657192	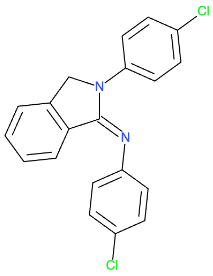
727192	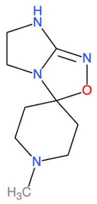	407820	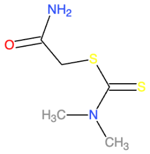

Table S1 Compounds that are super potent to cell lines that PECAN incorrectly predicted they were inactive to.

NSC ID	Structure	NSC ID	Structure
633713	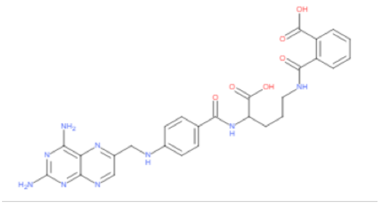	363981	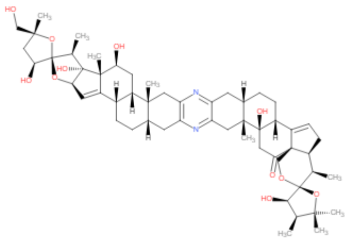
789804	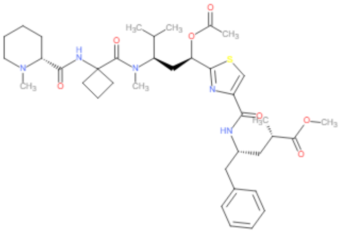	243023	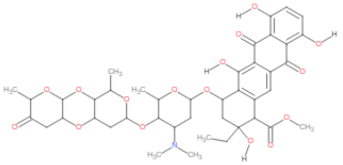
681638	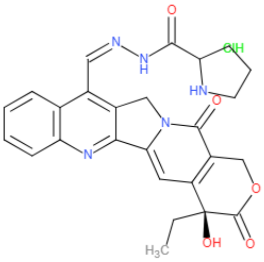	682309	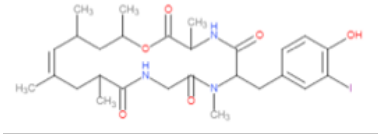
791458	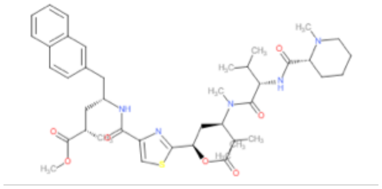	694330	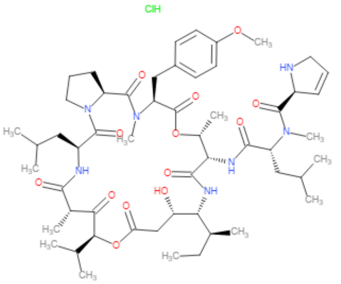

794717	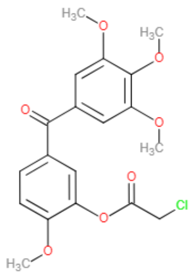	606699	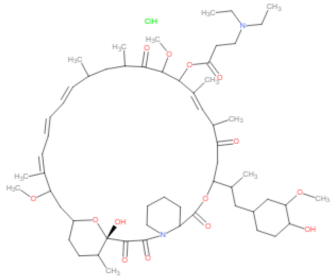
56030	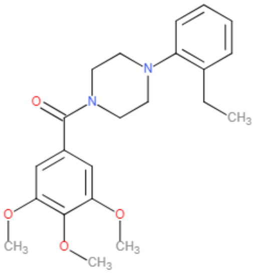	323241	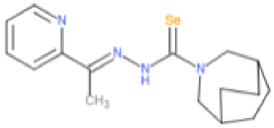
668329	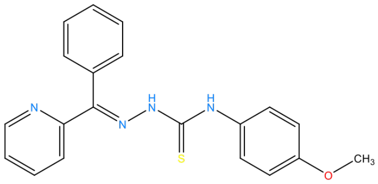	691041	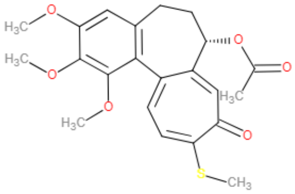
619029	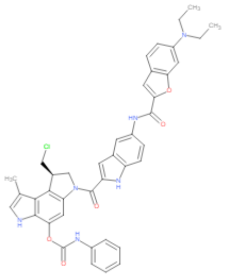	797907	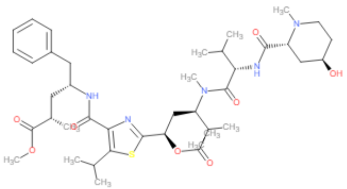

665804	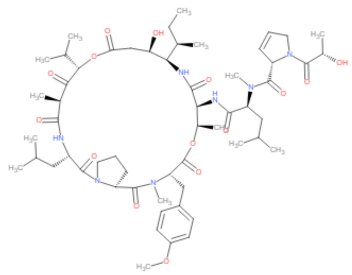	378736	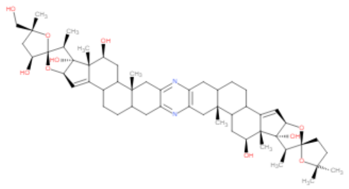
793137	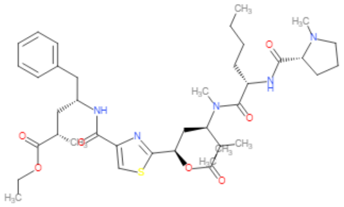	363980	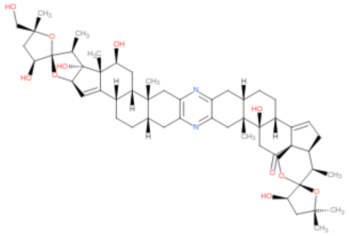
328785	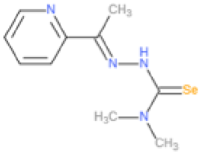	667642	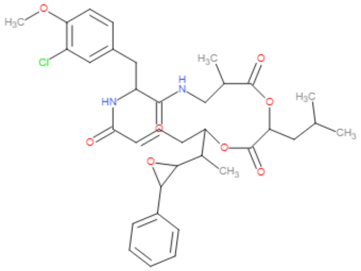
795963	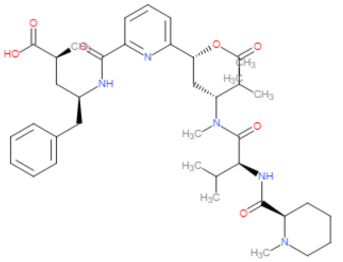	740645	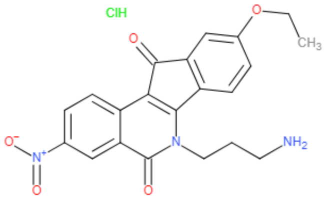

648780	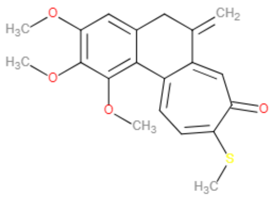	789800	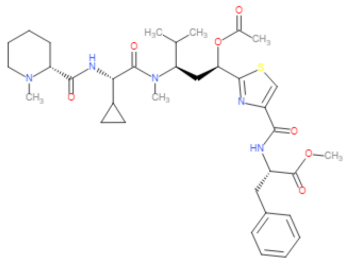
670121	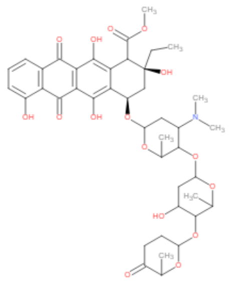	684362	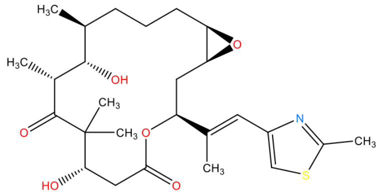
343493	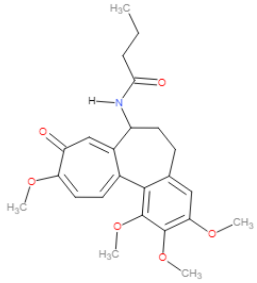	662161	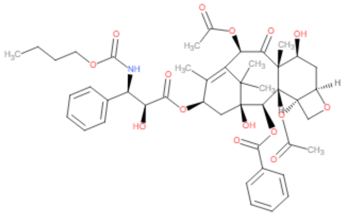
693540	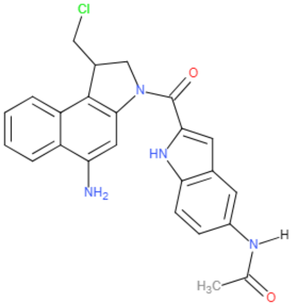	666608	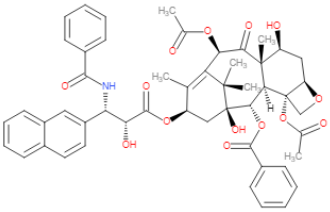

696075	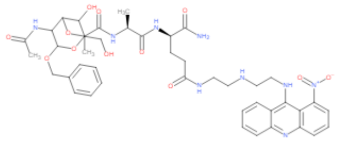	799253	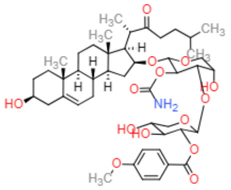
681641	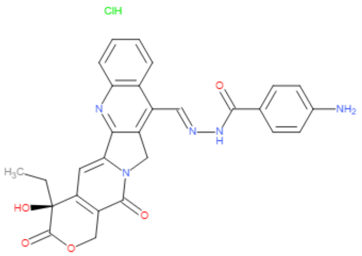	610458	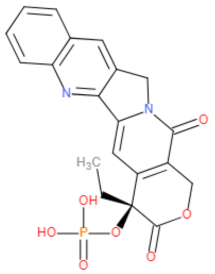
45384	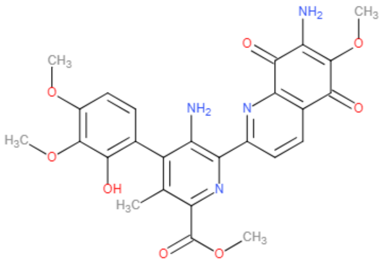	671034	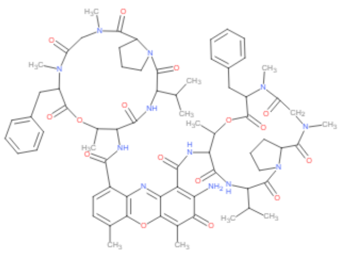
18335	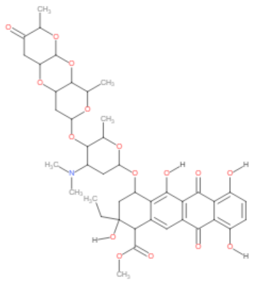	374998	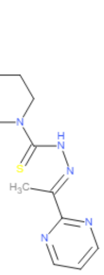

111533	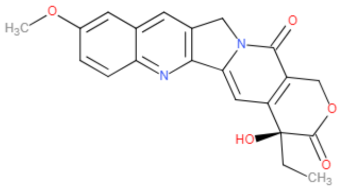	359708	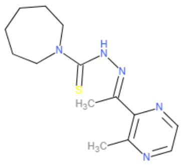
736989	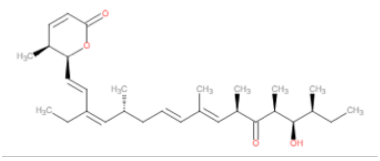	699490	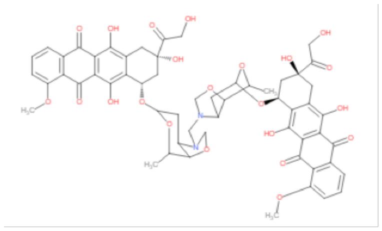
671035	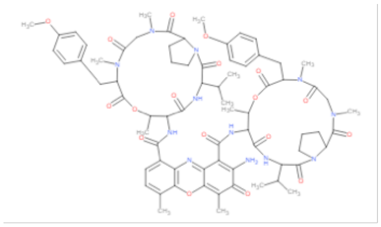	653244	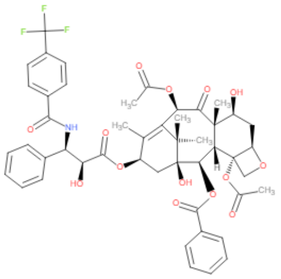
635450	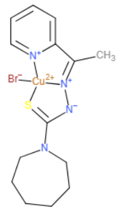	789808	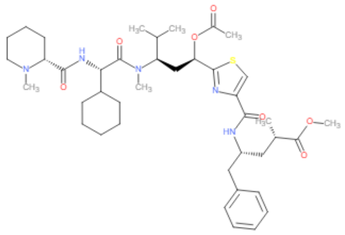

529861	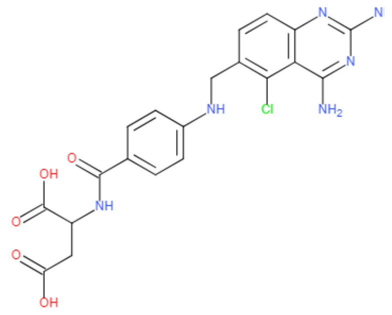	692219	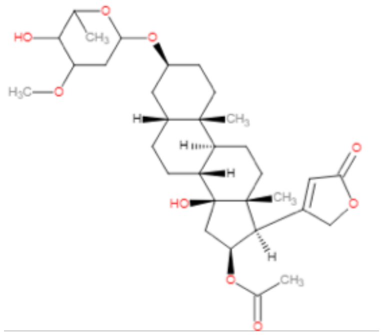
759174	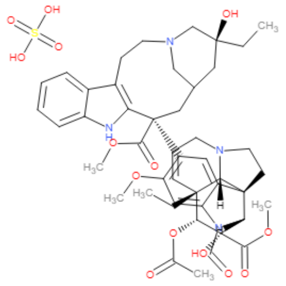	786094	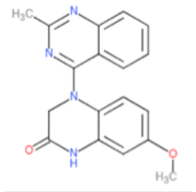
335789	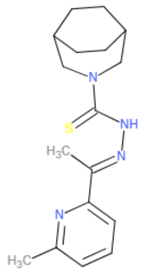	674622	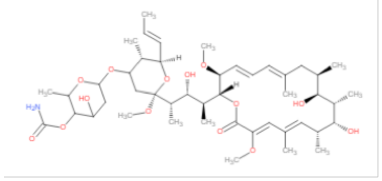
76919	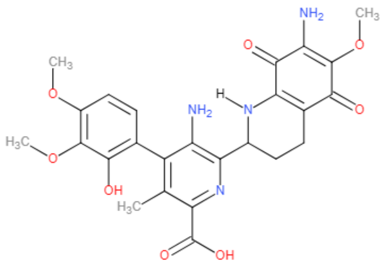		

Table S2 Compounds that are super potent to cell lines that PECAN correctly predicted they were super potent to.

NSC ID	Structure	NSC ID	Structure
122870	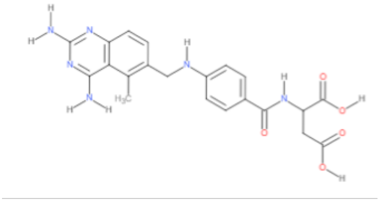	330753	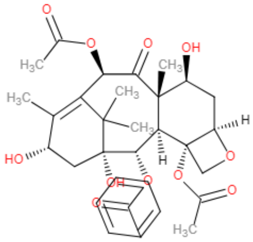
691041	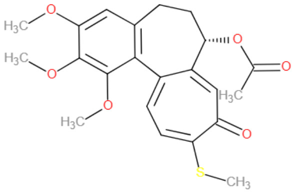	619029	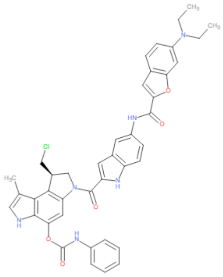
672141	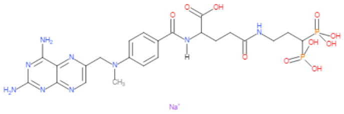	529861	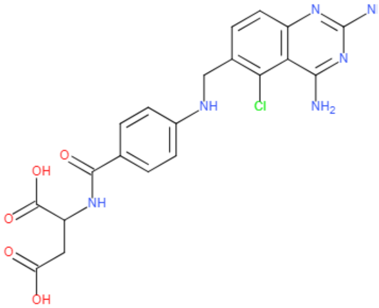

Table S3 Compounds that are inactive to cell lines that PECAN incorrectly predicted they were super potent to.

compounds from NPL-720 were excluded because experimental results on GI50 concentrations were not available. One remaining compound was excluded because the experimental results were inaccessible on the NCI database at the time of this analysis. The experimental data collected were processed in the same manner as the data in the training dataset. The 106 compounds used as an independent are described in Table S4. For each compound, the ID number, structure, CAS number, and NSC ID are given. We also include source information for the compounds.

Method S3. NPL-720 Test Set Results

Because each of the 106 compounds had been experimentally tested against a different set of 30-50 cell lines out of a total of 300 cell lines used by the NCI, we decided to use the average of all available experimental GI50 values as the experimental values to compare to our predictions. We then similarly averaged over our predictions to have one average GI50 value to compare to the experimental value. Therefore, each compound had an average cell line activity value.

The remainder of the analysis is the same as that done in section 3.2 of the main text. The accuracy of PECAN on NPL-720 is 57.55% and the within-one accuracy is 91.51%. We include the distribution of compounds across activity levels (Figure S1), a confusion matrix comparing PECAN predictions and true experimental labels (Figure S2), and a table with the precision and recall for each activity level (Figure S3). We note that the precision and recall values should be assessed keeping the size of the test set (106 compounds) in mind. Values appear as 0 for "active" and "super potent" activity levels because the NPL-720 test set includes 2 and 1 examples of these, respectively. With a larger test set, like that used in the main text (section 2.2), precision and recall values are more indicative of PECAN's true performance with compounds in these activity levels. PECAN's raw predictions of activity level for these 106 compounds on all 59 cell lines are provided in Table S5 and Table S6.

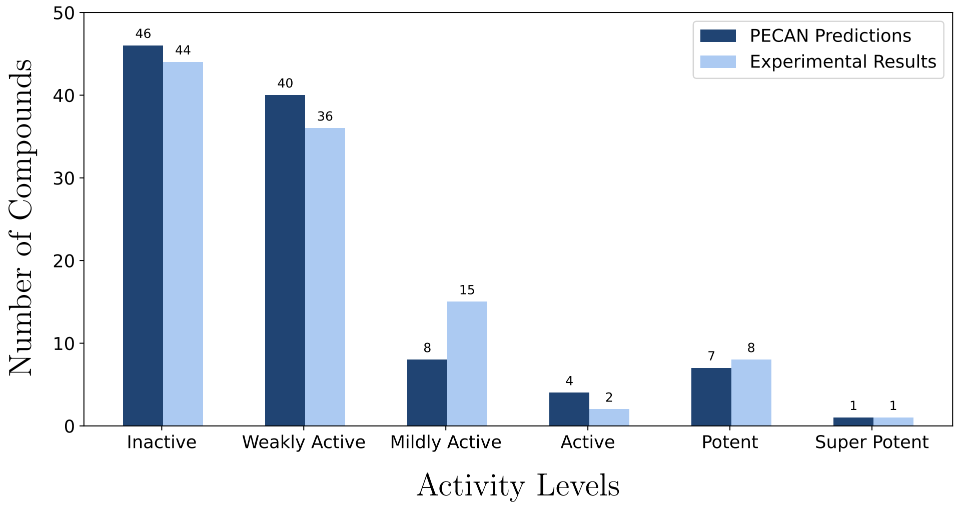

Figure S1 Counts of PECAN activity level predictions (averaged over cell line activities) and experimental activity levels.

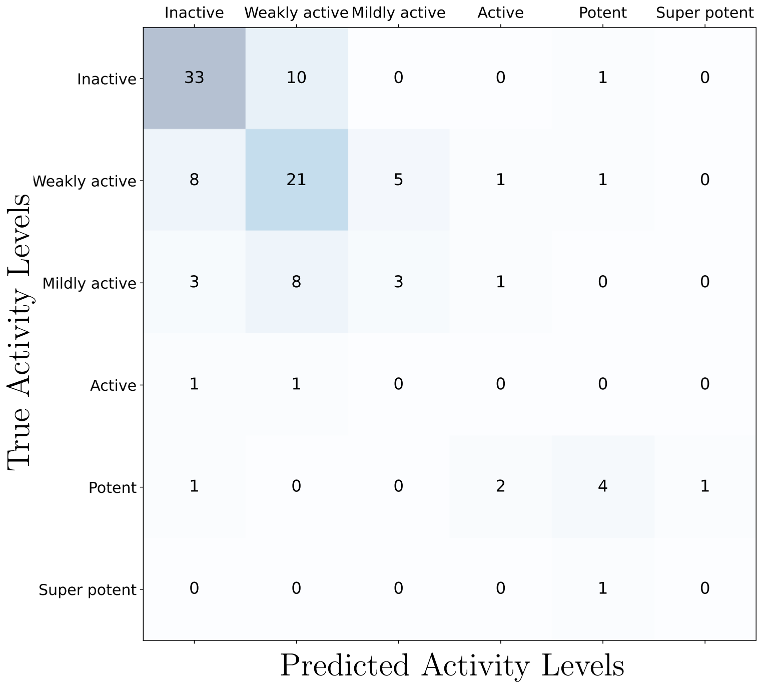

Figure S2 Confusion matrix for PECAN predictions. Columns indicate total predictions for each activity level, and rows indicate the true label for each prediction. Values on the diagonal indicate compounds that were correctly predicted (prediction matches label).

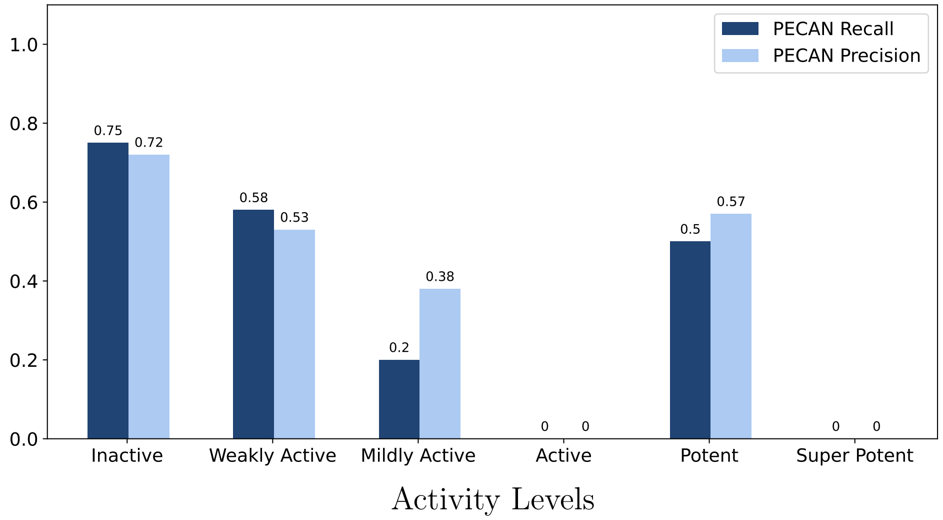

Figure S3 Recall and precision of PECAN predictions when compared with experimental results for 106 compounds from NPL-720 based on data shown in Figure S2.

	ID Number	Structure	CAS Number	NSC ID	Name	LogP	MW	Source
1	ST005174	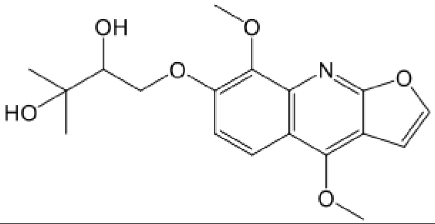	522-11-2	94653	Evoxine	1.58	347.37	From the leaves of Monnieria trifolia and the stem bark of Teclea gerrardii (Rutaceae: Toddalioideae).
2	ST008373	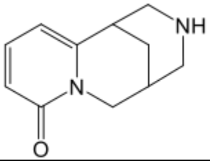	485-35-8	407282	Cytisine	-0.88	190.25	Seeds Laburnum Anagiroides and other Leguminosae.
3	ST013879	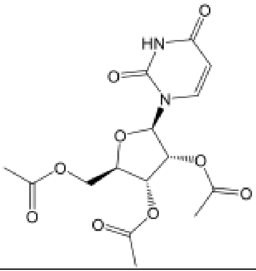	4105-38-8	788948	2',3',5'-Tri-O-acetyluridine	-1.59	370.32	Nucleoside analog.
4	ST018409	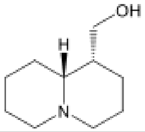	486-70-4	685529	Lupinine	0.94	169.27	Anabasis aphylla.

5	ST019369	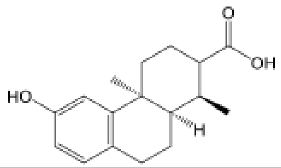	5947-49-9	231784	Podocarpic acid	3.91	274.36	Phytochemicals.
6	ST023509	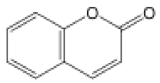	91-64-5	8774	Coumarin	1.82	146.15	Tonka Beans, lavander oil, woodruff, sweet clover, and numerous other plant sources.
7	ST023512	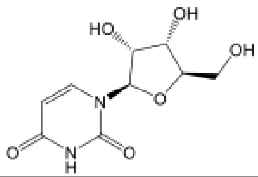	58-96-8	20256	Uridine; 1-beta-D-Ribo-furanosyluracil	-2.28	244.2	Nucleoside; widely distributed in nature.
8	ST023803	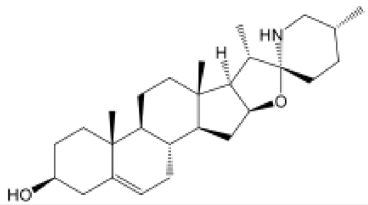	126-17-0	178260	Solasodine	4.95	413.65	Solanum lycocarpum St. Hil (Solanaceae) and fruit; Solanum laciniatum.
9	ST024713	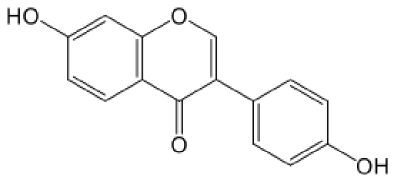	485-72-3	93360	Formononetin	2.13	254.24	Isoln from soy-bean meal (Soja hispida); from clover species Trifolium subterraneum L. and T. pratense L., and the major estrogenic factor in Leguminosae.

10	ST024752	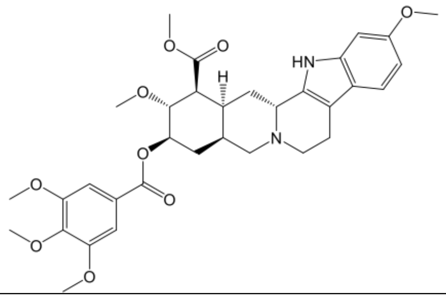	50-55-5	59272	Reserpine	2.69	608.69	Found in Rauwolfia spp.
11	ST024772	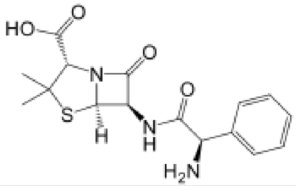	69-53-4	528986	Ampicillin	-.02	349.41	Semi-synthetic orally active antibiotic structurally related to penicillin. Amino derivative of penicillin.
12	ST028625	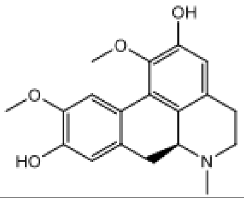	476-70-0	65689	Boldine	2.56	327.38	From boldo (Peumus boldus Molina, Monimiaceae); from Laurelia novaezelandiae.
13	ST040209	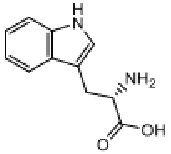	73-22-3	13119	L-Tryptophan	0.32	204.23	Precursor of serotonin; from casein.

14	ST041029	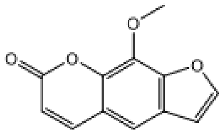	298-81-7	45923	8-Methoxypsoralen	1.31	216.19	Derivative of Psoralen, one of a group of furocoumarins occurring naturally in more than two dozen plant sources including Rutaceae (e.g. bergamot, limes, cloves), Umbelliferae (e.g. celery, parsnips), and Leguminosae (e.g. Psoralen coryfolia).
15	ST044516	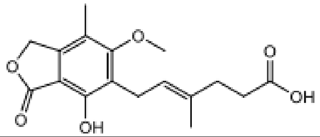	24280-93-1	129185	Mycophenolic acid	2.55	320.34	Penicillium brevi-compactum Dierckx.
16	ST052001	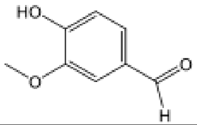	121-33-5	15351	Vanillin	1.27	152.15	Occurs naturally in a wide variety of foods and plants. Isolated from vanilla beans.
17	ST055352	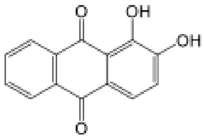	72-48-0	7212	Alizarin	1.64	240.21	Occurs in the root of the madder plant (Rubia tinctorum L., Rubiaceae; Krappwurzel).

18	ST055354	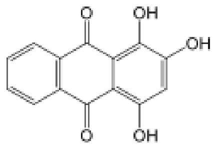	81-54-9	10447	Purpurin	1.25	256.21	Occurs as glycoside in the madder root (Rubia tinctorum L., Rubiaceae) of commerce. Formed during storage; no appreciable amount in the fresh root.
19	ST055355	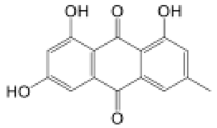	518-82-1	408120	Emodin	1.74	270.24	Occurs mostly as the rhamnoside (see Frangulin) in rhubarb root, alder buckthorn (Rhamnus frangula L.), Cascara sagrada (Rhamnus purshiana DC., Rhamnaceae), and in Rumex and other Polygonaceae; from rhubarb root and bark of alder buckthorn.
20	ST055359	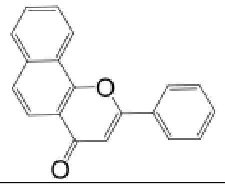	604-59-1	407011	7,8-Benzoflavone; alpha-Naphthoflavone	4.07	272.3	Flavonoid derivative.

21	ST055522	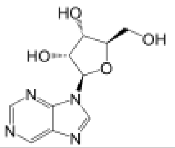	550-33-4	65423	Purine 9beta-D-ribofuranoside	-1.71	252.23	Nucleoside analog.
22	ST055629	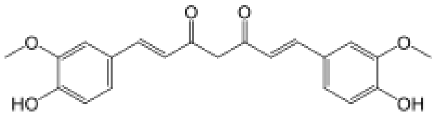	458-37-7	32982	Curcumine	2.56	368.39	Natural dyestuff from root of Curcuma longa L., Zingiberaceae.
23	ST055992	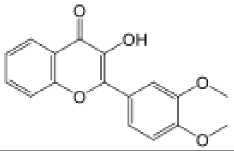	6889-80-1	102051	3-Hydroxy-3',4'-Dimethoxyflavone	1.66	298.29	Plant flavone derivative.
24	ST056186	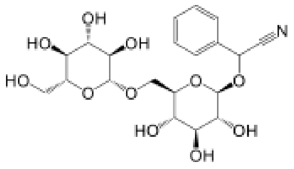	29883-15-6	15780	Amygdalin	2.13	457.43	From apricot kernels. Cynogenic glycoside which occurs in seeds of Rosaceae, principally in bitter almonds and in peaches and apricots.
25	ST056188	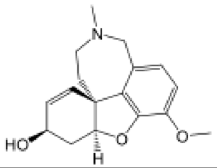	357-70-0	100058	(-) Galanthamine	1.41	287.36	Galanthus Krasnovii A. Chochr.
26	ST056220	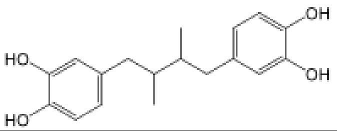	500-38-9	4291	Nordihydro-guaiaretic acid	4.48	302.37	From creosote bush, Larrea divaricata, Zygophyllaceae (Covillea tridentata).

27	ST056283	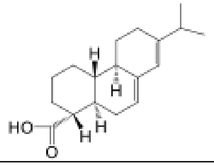	514-10-3	25149	Abietic acid	4.59	302.46	A widely available organic acid.
28	ST056285	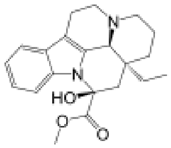	1617-90-9	91998	Vincamine	3.26	354.45	Major indole alkaloid of Vinca minor L., Apocynaceae occurring naturally in the d-form.
29	ST056287	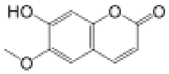	92-61-5	405647	Scopoletin	1.31	192.17	The aglucone of scopolin. Occurs in root of Scopolia japonica Maxim., Scopolia carniolica Jacq., Atropa belladonna L., Solanaceae, Convolvulus scammonia L., and Convolvulaceae.
30	ST056289	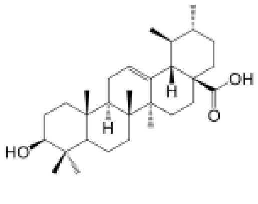	77-52-1	4060	Ursolic acid	7.33	456.71	Found in leaves and berries of Arctostaphylos uva-ursi (L.) Spreng (bearberry), Vaccinium macrocarpon Ait. (cranberry), Rhododendron hymenanthes Makino, and Ericaceae.

31	ST056292	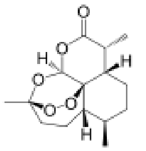	63968-64-9	369397	Artemisinin	3.17	282.34	From traditional Chinese medicinal herb Artemisia annua L., Compositae, which has been known as Qinghao.
32	ST056301	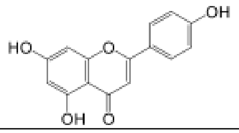	520-36-5	83244	Apigenin	1.9	270.24	The aglycon of linarin; flowers of Clerodendron infortunatum.
33	ST056305	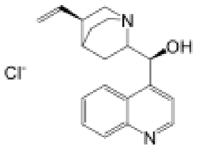	118-10-5	6176	(+)-Cinchonine	2.6	329.48	Occurs in most varieties of cinchona bark, especially in bark of Cinchona micrantha, Rubiaceae.
34	ST056306	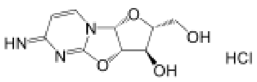	10212-25-6	145668	(-)-Cyclocytidine hydrochloride; Ancitabin hydrochloride	-0.21	261.66	Intermediate in the synthesis of cytarabine, which is a nucleoside analog.

35	ST056307	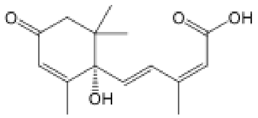	21293-29-8	146877	(±)-Abscisic acid, abscisin II; dormin; ABA	1.3	264.32	Abscission-accelerating plant hormone; naturally occurring as the (+)-cis, transform. From young cotton fruit and sycamore leaves. Identified in sycamore, birch, rose, cabbage, potato, lemon, and avocado.
36	ST056310	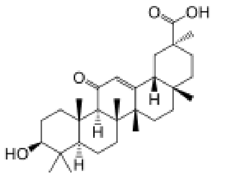	471-53-4	35347	Enoxolone	6.28	470.69	From glycyrrhizic acid, which is extracted from from Glycyrrhiza glabra L., Leguminosae.
37	ST056311	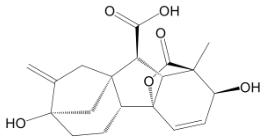	77-06-5	14190	Gibberellic Acid	0.45	346.38	Plant hormone; most outstanding of the plant-growth promoting metabolites of Gibberella fujikuroi.
38	ST056312	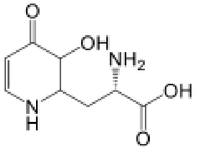	500-44-7	69188	L-Mimosine	-2.62	200.19	Naturally occurring amino acid; from Koa hoale seeds.

39	ST056341	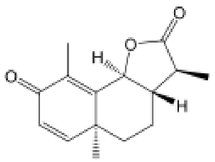	481-06-1	4900	a-Santonin	1.81	246.31	From the dried unexpanded flower heads of Artemisia maritima L., sens. lat., Compositae [Levant wormseed] and other species of Artemisia found principally in Russian and Chinese Turkestan and the Southern Ural region.
40	ST056348	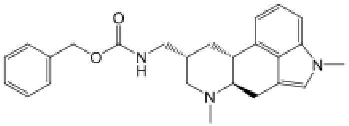	17692-51-2	755878	Metergoline phenylmethyl ester	3.42	403.53	Ergoline derivative.
41	ST056353	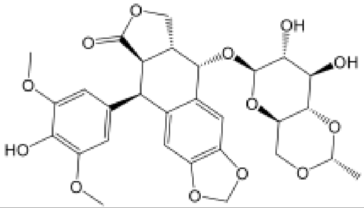	33419-42-0	141540	Etoposide; VP-16-213	1.12	558.56	Semi-synthetic derivative of podophyllotoxin.
42	ST056390	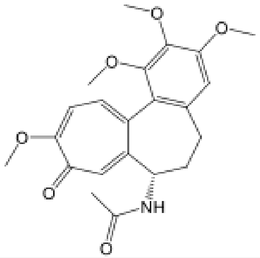	64-86-8	757	Colchicine	0.05	399.44	A major alkaloid of Colchicum autumnale L., Liliaceae.

43	ST056919	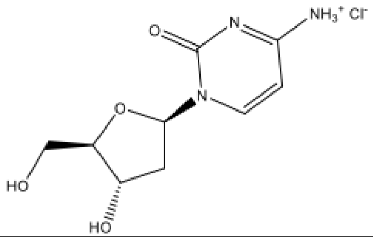	3992-42-5	83251	2’-Deoxycytidine hydrochloride	-1.23	262.67	Nucleoside, cytidine analog.
44	ST056939	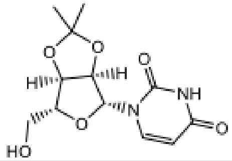	362-43-6	520038	2’,3’-O-Isopropyl-ideneuridine	-0.92	284.27	Nucleoside, uridine derivative.
45	ST056941	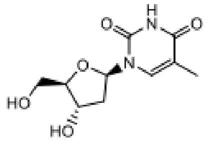	50-89-5	21548	Thymidine	-1.11	242.23	Constituent of deoxyribonucleic acid, which is an essential component of chromosomes in cell nuclei; from thymonucleic acid.
46	ST057073	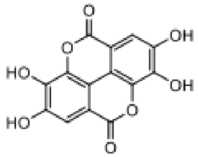	476-66-4	407286	Ellagic acid hydrate	1.05	302.19	From the kino of Eucalyptus maculata Hook and E. hemipholia F. Muell., Myrtaceae.
47	ST057075	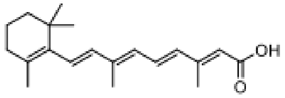	302-79-4	122758	all-trans-Retinoic acid; Tretinoin; vitamin A acid	1.45	300.44	Physiological metabolite of vitamin A.

48	ST057076	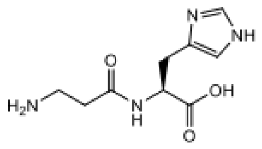	305-84-0	524045	L-Carnosine	-2.53	226.24	Naturally occurring dipeptide found in large amounts in skeletal muscle. Also present in other tissues such as brain, cardiac muscle, and kidney.
49	ST057091	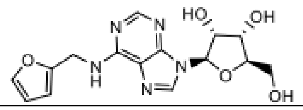	4338-47-0	120958	Kinetin-9-riboside	-1.15	347.33	Nucleoside analog.
50	ST057093	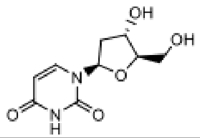	951-78-0	23615	2'-Deoxyuridine	-1.46	228.2	From an enzymatic hydrolysate of herring sperm DNA.
51	ST057095	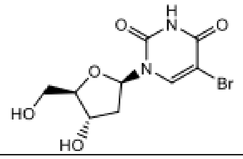	59-14-3	38297	5-Bromo-2'-deoxyuridine	1.16	307.1	Nucleoside analog.
52	ST057098	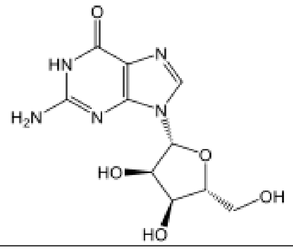	118-00-3	19994	Guanosine	-2.76	283.24	Nucleoside analog.

53	ST057163	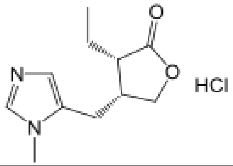	54-71-7	5746	Pilocarpine hydrochloride	0.54	244.72	Cholinergic principle from Pilocarpus jaborandi Holmes, Rutaceae.
54	ST057165	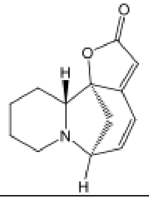	5610-40-2	107413	Securinine	0.54	217.27	From leaves and roots of Securinega suffruticosa, Euphorbiaceae found in the Ussuri region.
55	ST057166	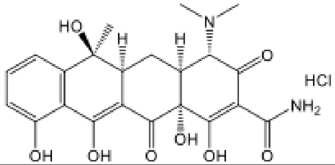	64-75-5	757338	Tetracycline hydrochloride	-3.26	480.9	Produced by Streptomyces spp.
56	ST057175	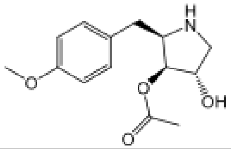	22862-76-6	76712	Anisomycin	0.64	265.31	From Streptomyces griseolus and S. roseochromogenes.
57	ST057182	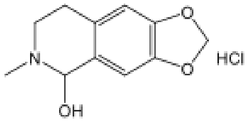	4884-68-8	93766	Hydrastinine hydrochloride	1.66	243.69	By oxidation of hydrastine, which is isolated from Hydrastis canadensis L., Ranunculaceae together with berberine and canadine.

58	ST057212	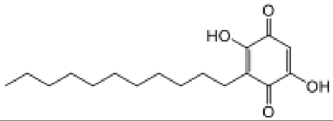	550-24-3	91874	Embelin	1.79	294.39	From fruit of Embelia ribes Burm., Myrsinaceae.
59	ST057225	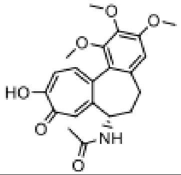	477-27-0	33411	Colchiceine	-0.31	385.41	Isolated from Colchicum autumnale L., Liliaceae.
60	ST057232	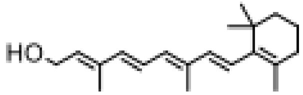	68-26-8	122759	Retinol; Vitamin A, synthetic, 95.%	4.69	286.46	Occurs pre-formed only in animals; metabolized from carotenoids, such as b-carotene, in the intestinal mucosa. Dietary sources include liver, milk, butter, cheese, eggs and fish liver oils or as carotenoids from fruits and vegetables.
61	ST057233	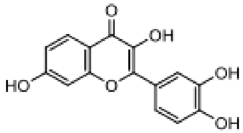	528-48-3	407010	Fisetin	0.78	286.23	Flavanoid present in the bark and stems of a variety of trees. Isolation from Rhus cotinus L. Anacardiaceae, Venetian Sumach; from heartwood of Acacia spp, Leguminosae.

62	ST057240	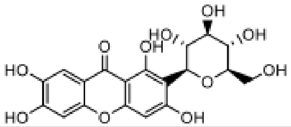	4773-96-0	248870	Mangiferin	-1.43	422.34	From Swertia chirata.
63	ST057251	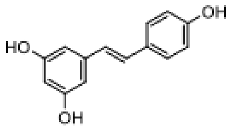	501-36-0	327430	Resveratrol, 99%	3.06	228.25	Phytoalexin found in a variety of plants; active ingredient of Asian folk medicine "Kojo-Kon", which is the powdered root of the Japanese knotweed; compound in red wine.
64	ST057253	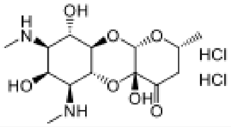	21736-83-4	248616	Spectinomycin	-2.32	405.27	Antibiotic isolated from fermentation broth of Streptomices Spectabilis.
65	ST057257	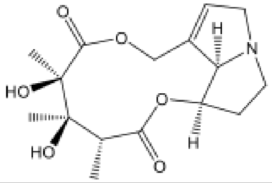	315-22-0	28693	Monocrotaline	-0.65	325.36	Toxic pyrrolizidine alkaloid isolated from Crotalaria spp.
66	ST057259	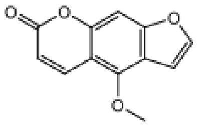	484-20-8	95437	Bergapten; 5-Methoxypsoralen, 99%	1.31	216.19	Naturally occurring analog of psoralen and isomer of methoxsalen found in a wide variety of plants.

67	ST057260	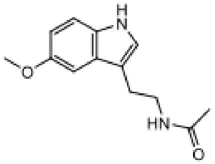	73-31-4	56423	Melatonin	0.71	232.28	A hormone of the pineal gland, also produced by extra-pineal tissues, that lightens skin color in amphibians by reversing the darkening effect of MSH; from the pineal glands of beef cattle.
68	ST057261	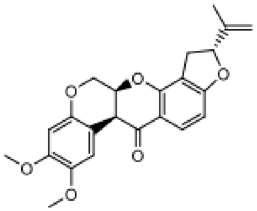	83-79-4	8505	Rotenone	2.9	394.42	Principal insecticidal constituent of derris root, cubé, etc.; from Lonchocarpus nicou (Aubl.) DC., Leguminosae.
69	ST057529	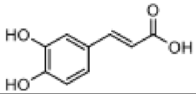	331-39-5	57197	Caffeic acid	1.15	180.16	From green coffee.
70	ST057531	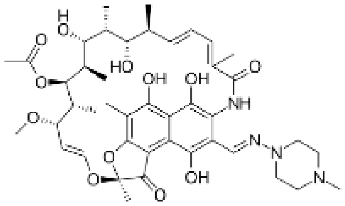	13292-46-1	113926	Rifampicin	2.7	822.95	Semisynthetic antibiotic obtained by reacting 3-formylrifamycin SV with 1-amino-4-methylpiperazine in tetrahydrofuran.
71	ST057580	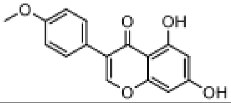	491-80-5	123538	Biochanin A	2.01	284.27	Found in red clover (Trifolium pratense).

72	ST057602	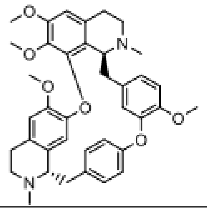	518-34-3	77037	(S,S)-(+)-Tetrandrine	6.39	622.76	From the root of Stephania tetrandra S. Moore, Menispermaceae.
73	ST057705	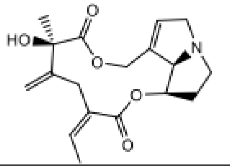	480-81-9	30622	Seneciphylline	0.59	333.38	Senecio species; e.g. Senecio bicolor.
74	ST057771	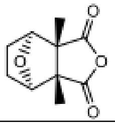	56-25-7	61805	Cantharidin	0.98	196.2	Active principle of cantharides and other insects; Spanish Fly aphrodisiac.
75	ST059837	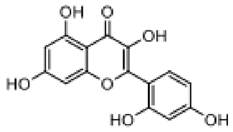	480-16-0	19801	Morin hydrate	0.35	302.24	In wood of old fustic (Chlorophora tinctoria (L.) Gaud., Moraceae), also called Cuba wood, or yellow Brazil wood. The wood of the Osage orange tree also contains morin.
76	ST066914	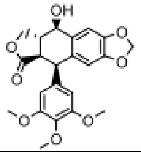	518-28-5	24818	Podophyllotoxin	2.12	414.41	Found in the rhizomes of North American Podophyllum peltatum L., Podophyllaceae.

77	ST069307	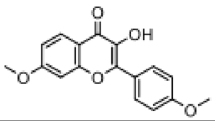	13198-99-7	102029	4’,7-Dimethoxy-3-hydroxyflavone	1.66	298.29	Plant flavone derivative.
78	ST069312	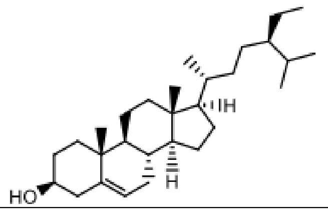	83-46-5	8096	beta-Sitosterol	8.14	414.72	Common sterol in plants. Isoln from wheat germ oil and corn oil.
79	ST069321	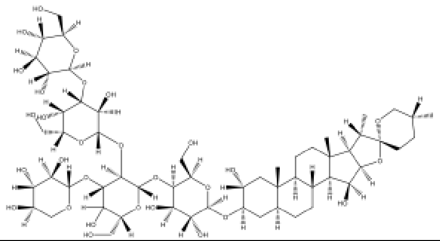	11024-24-1	23471	Digitonin	-6.5	1229.32	Obtained from the seeds of Digitalis purpurea L., Scrophulariaceae.
80	ST069324	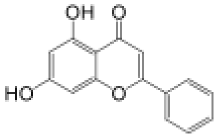	480-40-0	407436	Chrysin	2.29	254.24	From heartwood of Pinus monticola Dougl., P. excelsa Wall., and P. aristata Engelm., Pinaceae; from bark of Dolichandrone falcata Seem., Bisnomiaceae.

81	ST069327	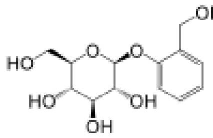	138-52-3	5751	D(-)-Salicin	-0.77	286.28	Usually obtained by making hot water extracts from the ground bark of poplar (Populus) and willow (Salix); also found in the leaves and female flowers of the willow; from root bark of Viburnum prunifolium L., Caprifoliaceae.
82	ST069329	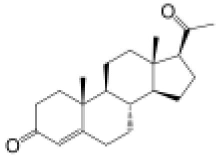	57-83-0	9704	Progesterone	3.78	314.47	Active principle of the corpus luteum, secreted during the latter half of the menstrual cycle; from corpus luteum of pregnant sows.
83	ST069334	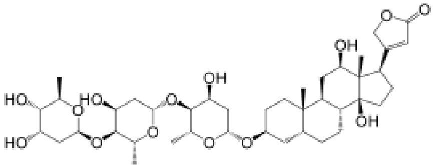	20830-75-5	95100	Digoxin	1.26	780.95	Secondary glycoside from Digitalis lanata Ehrh., or D. orientalis Lam., Scrophulariaceae.
84	ST069335	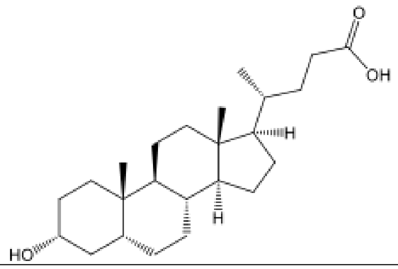	434-13-9	683770	Lithocholic acid	5.3	376.58	Found in ox bile, human bile, rabbit bile, and in ox and pig gallstones.

85	ST069355	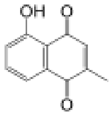	481-42-5	236613	Plumbagin	0.87	188.18	Found in the roots of Plumbago europaea L., P. zeylanica, P. rosea L., Plumbaginaceae.
86	ST069364	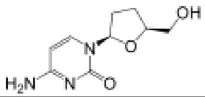	7481-89-2	606170	2’,3’-Dideoxycytidine	-0.58	211.22	Nucleoside derivative.
87	ST069381	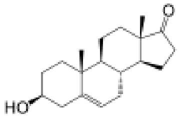	53-43-0	9896	trans-Dehydro-androsterone, dehydroisoadros-terone	3.71	288.43	Major secretory steroidal product of the adrenal gland; secretion progressively declines with aging.
88	ST072163	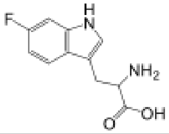	7730-20-3	9364	6-Fluoro-DL-tryptophan	0.48	222.22	Tryptophan derivative.
89	ST072172	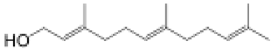	4602-84-0	60597	Farnesol	4.01	222.37	Found in oils of citronella, neroli, cyclamen, lemon grass, tuberose, rose, musk, balsam Peru, and tolu.
90	ST072187	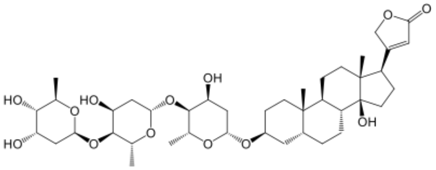	71-63-6	7529	Digitoxin	1.85	764.95	Secondary glycoside from Digitalis purpurea L., Scrophulariaceae. Extracted from the dried leaves.

91	ST073359	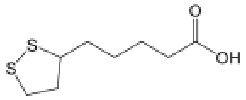	1077-28-7	90788	DL-Thioctic acid	2.08	206.32	Growth factor for many bacteria and protozoa; prosthetic group, coenzyme, or substrate in plants, microorganisms, and animal tissues.
92	ST075197	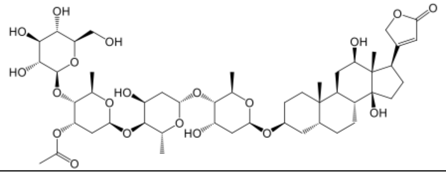	17575-22-3	7533	lanatoside C	0.61	985.13	Glycoside from the leaves of Digitalis lanata.
93	ST075201	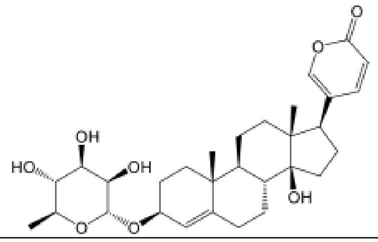	466-06-8	7521	proscillaridin A	2.3	530.65	Prepared by acid cleavage of scillaren A, which is from Roots of URGINEA INDICA K UNTH, (Liliaceae) Indian squill.
94	ST079379	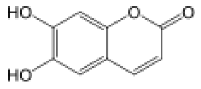	305-01-1	26428	Esculetin	1.05	178.14	Relatively widespread among angiosperm families: e.g., in the seeds of Euphorbia lathyris (Euphorbiaceae) and in the bark of Fraxinus spp.

95	ST085767	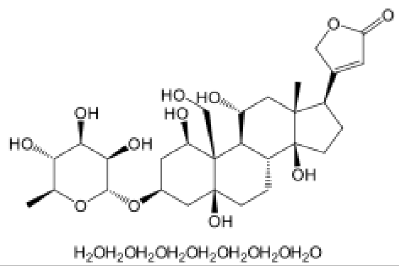	11018-89-6	757425	Ouabain octahydrate, G-Strophantin octahydrate	-2.0	728.78	Derivative of Ouabain, which is obtained from the seeds of Strophanthus gratus.
96	ST095786	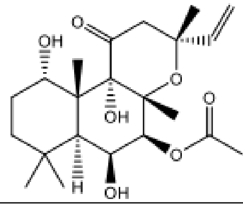	66575-29-9	375489	Forskolin	0.7	410.51	From Coleus forskohli.
97	ST098734	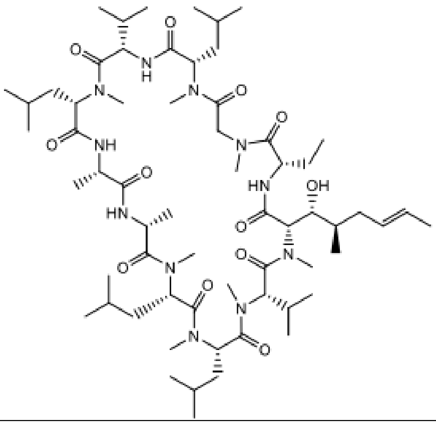	59865-13-3	290193	Cyclosporin A	3.64	1202.64	From Trichoderma polysporum (Link ex Pers.) Rifai.

98	ST098733	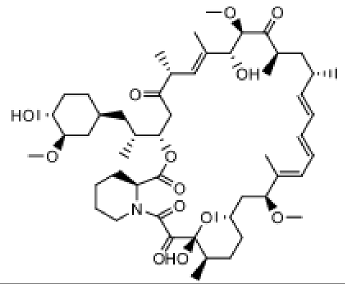	53123-88-9	226080	Rapamycin	4.3	914.19	Produced by Streptomyces hygroscopicus.
99	ST057235	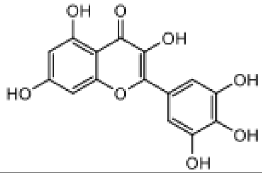	529-44-2	407290	Myricetin	-0.04	318.24	Black wattle tree leaves; Lysimachia vulgaris L.
100	ST065835	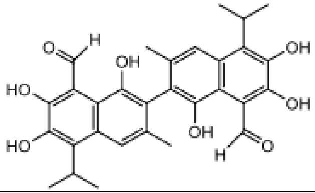	303-45-7	56817	Gossypol	6.3	518.56	From cotton seeds.
101	ST013858	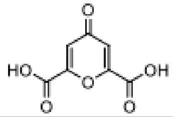	99-32-1	3979	Chelidonic acid	-2.26	184.1	Common in vegetables, Convallaria majalis L.
102	ST047334	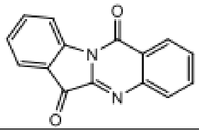	13220-57-0	349447	Tryptanthrine	2.27	248.24	From Candida lipolytica; from Isatis tinctoria.

103	ST092303	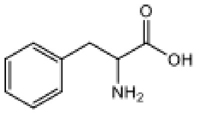	150-30-1	9959	DL-Phenylalanine, 99%	0.78	165.19	In some cereals; in buckwheat plants.
104	ST096004	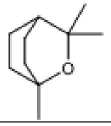	470-82-6	6171	Cineole; Eucalyptol	1.86	154.25	Found in essential oils and lavender oil.
105	ST097774	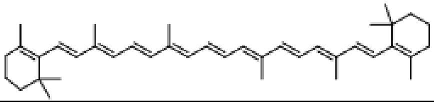	7235-40-7	62794	beta-Carotene	10.68	536.89	Plant carotenoid; in palm oil; present in liver.
106	ST056352	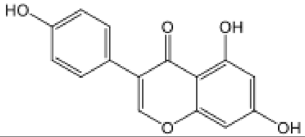	446-72-0	36586	Genistein, 4',5,7-Trihydroxyisoflavone	1.74	270.24	Found in soybean meal, in subterranean clover.

Table S4 ID number, structure, CAS number, NSC ID, name, LogP, molecular weight, and compound source for the 106 compounds in the external test set.

Despite only being able to compare predicted and experimental results on a subset of the compounds, NPL-720 offers avenues for further work in the identification of possible compounds for use in cancer chemotherapy. PECAN is designed to help speed drug discovery, and predictions on NPL-720 compounds whose experimental GI50 concentrations are not available could inform laboratory testing priorities. This work is currently underway.

Method S4. NPL-720 PECAN Predictions

Here we present the predictions of PECAN for the 106 compounds in the test set. For each compound, PECAN predicts the activity against 59 different cell lines. Predictions for cell lines 1 - 30 are listed in Table S5 and predictions for cell lines 31-59 are listed in Table S6. Each cell line and compound pair have a number, 0 through 5, as the predicted value for brevity. A prediction of 0 is a prediction of inactive, 1 indicates a prediction of weakly active, 2 indicates a prediction of mildly active, 3 indicates a prediction of active, 4 indicates a prediction of potent, and 5 indicates a prediction of super potent. The cell lines used are named in the tables for reference.

References

(1) TimTec Natural Product Library, 720. *https://www.timtec.net/* **2014**,

(2) National Cancer Institute, Developmental Therapeutics Program. *https://dtp.cancer.gov/dtpstandard /dwindex/index.jsp*

Library ID	Cell Lines																													
	DU-145	PC-3	HS 578T	MDA-MB-435	BT-549	MDA-MB-231/ATCC	T-47D	MCF 7	NCI-H460	NCI-H322M	EKVX	HOP-92	NCI-H23	A549/ATCC	NCI-H226	NCI-H522	HOP-62	SK-MEL-5	SK-MEL-2	UACC-257	SK-MEL-28	LOX IMVI	MALME-3M	M14	UACC-62	CAKI-1	786-0	UO-31	RXF 393	A498
ST005174	0	0	0	0	0	0	0	0	0	0	0	0	0	0	0	0	0	0	0	0	0	0	0	0	0	0	0	0	0	0
ST008373	0	1	1	1	1	1	1	1	1	0	1	1	1	1	1	1	1	1	1	1	0	1	1	1	1	0	1	1	1	1
ST013879	0	0	0	0	0	0	0	0	0	0	0	0	0	0	0	0	0	0	0	0	0	0	0	0	0	0	0	0	0	0
ST018409	0	0	0	0	0	0	0	0	0	0	0	0	0	0	0	0	0	0	0	0	0	0	0	0	0	0	0	0	0	0
ST019369	0	0	0	0	0	0	0	0	0	0	0	1	0	0	0	0	0	0	0	0	0	0	0	0	0	0	0	0	0	0
ST023509	0	0	0	0	0	0	0	0	0	0	0	0	0	0	0	0	0	0	0	0	0	0	0	0	0	0	0	0	0	0
ST023512	0	0	0	0	0	0	0	0	0	0	0	0	0	0	0	0	0	0	0	0	0	0	0	0	0	0	0	0	0	0
ST023803	1	1	1	1	1	1	2	1	1	1	1	1	1	1	1	1	1	1	1	1	1	1	1	1	1	1	1	1	1	1
ST024713	1	1	1	1	1	1	2	1	1	1	1	1	1	1	1	1	1	1	1	1	1	1	1	1	1	1	1	1	1	1
ST024752	2	2	2	2	1	1	2	2	2	1	1	2	1	1	1	2	1	1	1	1	1	2	1	2	2	1	2	2	2	1
ST024772	0	0	0	0	0	0	0	0	0	0	0	0	0	0	0	0	0	0	0	0	0	0	0	0	0	0	0	0	0	0
ST028625	1	1	1	1	1	1	1	1	1	0	1	1	1	1	1	1	1	1	1	1	1	1	1	1	1	1	1	1	1	1
ST040209	0	0	0	0	0	0	0	0	0	0	0	0	0	0	0	0	0	0	0	0	0	0	0	0	0	0	0	0	0	0
ST041029	0	0	0	0	0	0	0	0	0	0	0	0	0	0	0	0	0	0	0	0	0	0	0	0	0	0	0	0	0	0
ST044516	3	3	3	3	3	3	3	3	4	3	1	3	3	3	3	3	3	3	3	1	3	3	3	3	3	3	3	3	3	3
ST052001	0	0	0	0	0	0	0	0	0	0	0	0	0	0	0	0	0	0	0	0	0	0	0	0	0	0	0	0	0	0

ST055352	1	1	1	1	1	1	2	1	1	1	1	1	1	1	1	2	1	1	1	1	1	1	1	1	1	1	1	1	1	1
ST055354	1	1	1	1	1	1	1	1	1	1	1	1	1	1	1	1	1	1	1	1	1	1	1	1	1	1	1	1	1	1
ST055355	0	1	1	1	1	1	1	1	1	0	1	1	1	1	1	1	0	1	0	0	0	1	1	0	1	0	1	1	1	1
ST055359	0	1	1	1	1	1	1	1	1	0	1	1	1	0	1	1	0	1	0	0	0	1	0	0	1	0	1	1	1	0
ST055522	0	0	0	0	0	0	0	0	0	0	0	0	0	0	0	0	0	0	0	0	0	0	0	0	0	0	0	0	0	0
ST055629	1	1	1	1	1	1	1	1	1	1	1	1	1	1	1	1	1	1	1	1	1	1	1	1	1	1	1	1	1	1
ST055992	2	2	2	2	2	2	2	2	2	1	1	2	2	2	1	2	1	2	1	1	1	2	2	2	2	2	2	2	2	1
ST056186	0	0	0	0	0	0	0	0	0	0	0	0	0	0	0	0	0	0	0	0	0	0	0	0	0	0	0	0	0	0
ST056188	0	0	0	0	0	0	0	0	0	0	0	0	0	0	0	0	0	0	0	0	0	0	0	0	0	0	0	0	0	0
ST056220	1	1	1	1	1	1	1	1	1	1	1	1	1	1	1	1	1	1	1	1	1	1	1	1	1	1	1	1	1	1
ST056283	1	1	1	1	1	1	1	1	1	1	1	1	1	1	1	1	1	1	1	1	1	1	1	1	1	1	1	1	1	1
ST056285	1	1	1	1	1	1	1	1	1	1	1	1	1	1	1	1	1	1	1	1	1	1	1	1	1	1	1	1	1	1
ST056287	0	0	0	0	0	0	0	0	0	0	0	0	0	0	0	0	0	0	0	0	0	0	0	0	0	0	0	0	0	0
ST056289	1	1	1	1	1	1	1	1	1	1	1	1	1	1	1	1	1	1	1	1	1	1	1	1	1	1	1	1	1	1
ST056292	4	5	4	4	4	4	5	5	5	4	4	4	4	4	4	4	4	4	4	4	4	5	4	4	4	4	4	4	4	4
ST056301	1	1	1	1	1	1	1	1	1	1	1	1	1	1	1	1	1	1	1	1	1	1	1	1	1	1	1	1	1	1
ST056305	1	1	1	1	1	1	1	1	1	1	1	1	1	1	1	1	1	1	1	1	1	1	1	1	1	1	1	1	1	1
ST056306	2	2	2	3	2	2	3	3	3	2	2	3	2	2	2	3	2	3	2	2	2	3	2	2	3	3	2	2	3	2
ST056307	0	0	0	0	0	0	0	0	0	0	0	0	0	0	0	0	0	0	0	0	0	0	0	0	0	0	0	0	0	0
ST056311	0	0	0	0	0	0	0	0	0	0	0	0	0	0	0	0	0	0	0	0	0	0	0	0	0	0	0	0	0	0
ST056310	1	1	1	1	1	1	2	1	1	1	1	1	1	1	1	1	1	1	1	1	1	1	1	1	1	1	1	1	1	1
ST056312	0	0	0	0	0	0	0	0	0	0	0	0	0	0	0	0	0	0	0	0	0	0	0	0	0	0	0	0	0	0

ST056341	0	1	1	1	0	1	1	1	1	0	1	1	1	0	0	1	0	1	0	0	0	1	0	0	1	0	1	1	1	1
ST056348	0	0	1	0	0	0	0	1	0	0	0	1	0	0	0	1	0	1	0	0	0	0	0	0	0	0	0	0	1	0
ST056353	3	3	3	3	3	3	3	4	4	3	2	3	3	4	3	3	4	3	3	3	3	3	3	3	3	3	3	3	3	3
ST056390	4	4	4	5	4	4	4	4	4	4	4	4	4	4	4	4	4	4	4	4	4	4	4	4	4	4	4	4	4	4
ST056919	0	0	0	0	0	0	0	0	0	0	0	0	0	0	0	0	0	0	0	0	0	0	0	0	0	0	0	0	0	0
ST056939	0	0	0	0	0	0	0	0	0	0	0	0	0	0	0	0	0	0	0	0	0	0	0	0	0	0	0	0	0	0
ST056941	0	0	0	0	0	0	0	0	0	0	0	0	0	0	0	0	0	0	0	0	0	0	0	0	0	0	0	0	0	0
ST057073	1	1	1	1	1	1	1	1	1	0	1	1	1	1	1	1	1	1	1	1	1	1	1	1	1	1	1	1	1	1
ST057075	0	0	0	0	0	0	0	0	0	0	0	0	0	0	0	0	0	0	0	0	0	0	0	0	0	0	0	0	0	0
ST057076	0	0	0	0	0	0	0	0	0	0	0	0	0	0	0	0	0	0	0	0	0	0	0	0	0	0	0	0	0	0
ST057091	2	0	2	0	2	2	0	2	0	0	0	0	0	0	0	3	0	0	0	0	0	0	0	0	0	3	0	0	0	0
ST057093	0	0	0	0	0	0	0	0	0	0	0	0	0	0	0	0	0	0	0	0	0	0	0	0	0	0	0	0	0	0
ST057095	0	0	0	0	0	0	0	0	0	0	0	0	0	0	0	0	0	0	0	0	0	0	0	0	0	0	0	0	0	0
ST057098	0	0	0	0	0	0	0	0	0	0	0	0	0	0	0	0	0	0	0	0	0	0	0	0	0	0	0	0	0	0
ST057163	0	0	0	0	0	0	0	0	0	0	0	0	0	0	0	0	0	0	0	0	0	0	0	0	0	0	0	0	0	0
ST057165	1	1	1	1	1	1	1	1	1	1	1	1	1	1	1	1	1	1	1	1	1	1	1	1	1	1	1	1	1	1
ST057172	1	1	1	1	1	1	1	1	1	1	1	1	1	1	1	1	1	1	1	1	1	1	1	1	1	1	1	1	1	1
ST057175	0	0	0	0	0	0	0	0	0	0	0	0	0	0	0	0	0	0	0	0	0	0	0	0	0	0	0	0	0	0
ST057182	0	0	0	0	0	0	0	0	0	0	0	0	0	0	0	0	0	0	0	0	0	0	0	0	0	0	0	0	0	0
ST057212	1	1	1	2	1	1	2	1	1	1	1	1	1	1	1	2	1	1	1	1	1	2	1	1	1	2	1	1	1	1
ST057225	4	4	4	5	4	4	4	4	4	4	4	4	4	4	4	4	4	4	4	4	4	4	4	4	4	4	4	4	4	4
ST057232	0	0	1	1	0	0	0	1	0	0	0	1	0	0	0	1	0	1	0	0	0	1	0	0	0	0	0	0	1	0

ST057233	1	1	1	1	1	1	1	1	1	1	1	1	1	1	1	1	1	1	1	1	1	1	1	1	1	1	1	1	1	1
ST057240	0	0	0	0	0	0	0	0	0	0	0	0	0	0	0	0	0	0	0	0	0	0	0	0	0	0	0	0	0	0
ST057251	2	2	2	2	2	1	2	2	2	1	1	2	1	1	1	2	1	2	1	1	1	2	1	2	2	2	1	2	2	1
ST057253	0	0	0	0	0	0	0	0	0	0	0	0	0	0	0	0	0	0	0	0	0	0	0	0	0	0	0	0	0	0
ST057257	0	0	0	0	0	0	0	0	0	0	0	0	0	0	0	0	0	0	0	0	0	0	0	0	0	0	0	0	0	0
ST057259	0	0	0	0	0	0	0	0	0	0	0	0	0	0	0	0	0	0	0	0	0	0	0	0	0	0	0	0	0	0
ST057260	0	0	0	0	0	0	0	0	0	0	0	0	0	0	0	0	0	0	0	0	0	0	0	0	0	0	0	0	0	0
ST057261	0	1	1	1	1	1	1	1	1	0	1	1	1	1	1	1	0	1	1	0	0	1	0	0	1	0	1	1	1	1
ST057529	0	0	0	0	0	0	0	0	0	0	0	0	0	0	0	0	0	0	0	0	0	0	0	0	0	0	0	0	0	0
ST057531	2	2	2	2	2	2	2	2	2	2	2	2	2	2	2	2	2	2	2	2	2	2	2	2	2	2	2	2	2	2
ST057580	1	1	1	1	1	1	2	1	1	1	1	1	1	1	1	1	1	1	1	1	1	1	1	1	1	1	1	1	1	1
ST057602	1	2	1	2	1	1	2	2	1	1	1	2	1	1	1	2	1	1	1	1	1	2	1	1	1	1	1	1	2	1
ST057705	1	1	1	1	1	1	1	1	1	0	1	1	1	1	1	1	1	1	1	1	1	1	1	1	1	1	1	1	1	1
ST057771	0	0	0	0	0	0	0	0	0	0	0	0	0	0	0	0	0	0	0	0	0	0	0	0	0	0	0	0	0	0
ST059837	1	1	1	1	1	1	1	1	1	1	1	1	1	1	1	1	1	1	1	1	1	1	1	1	1	1	1	1	1	1
ST066914	3	3	3	3	3	3	3	3	3	3	3	3	3	3	3	3	3	3	3	3	3	3	3	3	3	3	3	3	3	3
ST069307	1	1	1	1	1	1	2	1	1	1	1	1	1	1	1	1	1	1	1	1	1	1	1	1	1	1	1	1	1	1
ST069312	1	1	1	1	1	1	1	1	1	1	1	1	1	1	1	1	1	1	1	1	1	1	1	1	1	1	1	1	1	1
ST069321	2	2	2	2	2	2	2	3	3	2	2	2	2	2	2	3	2	2	2	2	2	3	2	2	2	2	2	2	2	2
ST069324	1	1	1	1	1	1	1	1	1	1	1	1	1	1	1	1	1	1	1	1	1	1	1	1	1	1	1	1	1	1
ST069327	0	0	0	0	0	0	0	0	0	0	0	0	0	0	0	0	0	0	0	0	0	0	0	0	0	0	0	0	0	0
ST069329	1	1	1	1	1	1	1	1	1	1	1	1	1	1	1	1	1	1	1	1	1	1	1	1	1	1	1	1	1	1

ST069334	4	4	4	4	4	4	4	4	4	4	4	4	4	4	4	5	4	4	4	4	4	4	4	4	4	4	4	4	4	4
ST069335	0	1	1	1	1	1	1	1	1	0	1	1	1	1	1	1	1	1	1	0	0	1	1	1	1	0	1	1	1	1
ST069355	2	2	2	2	2	2	2	2	2	1	1	2	2	1	1	2	1	2	1	2	2	2	2	2	2	2	2	2	2	1
ST069364	0	0	0	0	0	0	0	0	0	0	0	0	0	0	0	0	0	0	0	0	0	0	0	0	0	0	0	0	0	0
ST069381	0	1	1	1	1	1	1	1	1	0	1	1	1	1	1	1	0	1	1	1	0	1	0	1	1	1	1	1	1	1
ST072163	0	0	0	0	0	0	0	0	0	0	0	0	0	0	0	0	0	0	0	0	0	0	0	0	0	0	0	0	0	0
ST072172	0	0	0	0	0	0	0	0	0	0	0	0	0	0	0	0	0	0	0	0	0	0	0	0	0	0	0	0	0	0
ST072187	5	4	4	4	4	4	4	5	5	4	4	4	4	5	4	5	5	4	4	4	4	5	4	4	4	4	5	4	4	4
ST073359	0	0	0	0	0	0	0	0	0	0	0	0	0	0	0	0	0	0	0	0	0	0	0	0	0	0	0	0	0	0
ST075197	4	4	4	4	4	4	4	4	4	4	4	4	4	4	4	5	4	4	4	4	4	4	4	4	4	4	4	4	4	4
ST075201	4	4	4	4	4	4	4	4	4	4	4	4	4	4	4	4	4	4	4	4	4	4	4	4	4	4	4	4	4	4
ST079379	0	0	0	0	0	0	0	0	0	0	0	0	0	0	0	0	0	0	0	0	0	0	0	0	0	0	0	0	0	0
ST085767	4	3	2	2	3	3	3	5	5	2	2	4	4	2	4	3	5	3	2	3	2	4	3	3	3	4	4	4	4	4
ST095786	0	1	1	1	0	1	1	1	1	0	1	1	0	0	0	1	0	1	0	0	0	1	0	0	1	0	1	1	1	0
ST098734	1	1	1	1	1	1	1	1	1	0	1	1	1	1	1	1	1	1	1	1	0	1	1	1	1	1	1	1	1	1
ST098733	4	5	5	5	5	5	5	5	5	5	5	5	4	5	5	5	5	5	5	5	5	5	5	5	5	5	5	5	5	5
ST057235	1	1	1	1	1	1	1	1	1	1	1	1	1	1	1	1	1	1	1	1	1	1	1	1	1	1	1	1	1	1
ST065835	2	2	2	2	2	2	2	2	2	2	2	2	2	2	2	2	2	2	2	2	2	2	2	2	2	2	2	2	2	2
ST047334	0	1	1	1	1	1	1	1	1	0	1	1	1	1	1	1	1	1	1	0	0	1	1	1	1	0	1	1	1	1
ST092303	0	0	0	0	0	0	0	0	0	0	0	0	0	0	0	0	0	0	0	0	0	0	0	0	0	0	0	0	0	0
ST095415	0	0	0	0	0	0	0	0	0	0	0	0	0	0	0	0	0	0	0	0	0	0	0	0	0	0	0	0	0	0
ST096004	0	0	0	0	0	0	0	1	0	0	0	1	0	0	0	0	0	1	0	0	0	0	0	0	0	0	0	0	1	0

ST097774	0	1	1	1	1	1	1	1	1	0	1	1	1	0	1	1	0	1	0	0	0	1	0	0	1	0	1	1	1	1
ST056352	1	1	1	1	1	1	2	1	1	1	1	1	1	1	1	1	1	1	1	1	1	1	1	1	1	1	1	1	1	1

Table S5 Predictions of antiproliferative activity of select NPL-720 compounds (the 106 with available experimental data) against the first 30 cell lines the network was trained on.

Library ID	Cell Lines																											
	SN12C	TK-10	ACHN	HCT-15	HCC-2998	COLO 205	KM12	HCT-116	SW-620	HT29	SNB-75	SF-295	SF-268	SF-539	U251	SNB-19	RPMI-8226	HL-60	CCRF-CEM	SR	MOLT-4	K-562	OVCAR-3	OVCAR-4	IGROV1	OVCAR-5	SK-OV-3	OVCAR-8	NCI/ADR-RES
ST005174	0	0	0	0	0	0	0	0	0	0	0	0	0	0	0	0	0	0	0	0	0	0	0	0	0	0	0	0	0
ST008373	0	0	1	1	0	1	1	1	1	1	1	0	1	1	1	0	1	1	1	1	1	1	1	1	1	0	0	1	1
ST013879	0	0	0	0	0	0	0	0	0	0	0	0	0	0	0	0	0	0	0	0	0	0	0	0	0	0	0	0	0
ST018409	0	0	0	0	0	0	0	0	0	0	0	0	0	0	0	0	0	0	0	0	0	0	0	0	0	0	0	0	0
ST019369	0	0	0	0	0	0	0	0	0	0	1	0	0	0	0	0	0	0	0	0	0	0	0	0	0	0	0	0	0
ST023509	0	0	0	0	0	0	0	0	0	0	0	0	0	0	0	0	0	0	0	0	0	0	0	0	0	0	0	0	0
ST023512	0	0	0	0	0	0	0	0	0	0	0	0	0	0	0	0	0	0	0	0	0	0	0	0	0	0	0	0	0
ST023803	1	1	1	1	1	1	1	1	1	1	1	1	1	1	1	1	2	2	2	2	2	2	1	1	1	1	1	1	1
ST024713	1	1	1	1	1	1	1	1	1	1	1	1	1	1	1	1	2	2	2	2	2	2	1	1	1	1	1	1	1
ST024752	1	1	2	2	2	1	2	2	2	2	2	2	1	1	2	1	2	2	2	2	2	2	2	2	2	1	1	1	2
ST024772	0	0	0	0	0	0	0	0	0	0	0	0	0	0	0	0	0	0	0	0	0	0	0	0	0	0	0	0	0
ST028625	1	1	1	1	1	1	1	1	1	1	1	1	1	1	1	1	1	1	1	1	1	1	1	1	1	1	0	1	1
ST040209	0	0	0	0	0	0	0	0	0	0	0	0	0	0	0	0	0	0	0	0	0	0	0	0	0	0	0	0	0
ST041029	0	0	0	0	0	0	0	0	0	0	0	0	0	0	0	0	0	0	0	0	0	0	0	0	0	0	0	0	0
ST044516	3	1	3	3	3	3	3	3	3	3	3	3	3	3	3	3	3	3	3	4	3	3	3	2	3	2	3	3	3
ST052001	0	0	0	0	0	0	0	0	0	0	0	0	0	0	0	0	0	0	0	0	0	0	0	0	0	0	0	0	0
ST055352	1	1	1	1	1	1	1	1	1	1	1	1	1	1	1	1	2	2	2	2	2	2	1	1	1	1	1	1	1

ST055354	1	1	1	1	1	1	1	1	1	1	1	1	1	1	1	1	1	2	1	2	1	1	1	1	1	1	1	1	1
ST055355	0	0	0	1	0	1	1	1	0	1	1	0	1	1	1	0	1	1	1	1	1	1	1	1	0	0	0	1	1
ST055359	0	0	1	1	0	0	0	1	0	1	1	0	1	1	1	0	1	1	1	1	1	1	0	1	0	0	0	1	1
ST055522	0	0	0	0	0	0	0	0	0	0	0	0	0	0	0	0	0	0	0	0	0	0	0	0	0	0	0	0	0
ST055629	1	1	1	1	1	1	1	1	1	1	1	1	1	1	1	1	1	1	1	1	1	1	1	1	1	1	1	1	1
ST055992	2	1	2	2	2	2	2	2	2	2	2	1	2	2	2	1	2	2	2	2	2	2	2	2	2	1	1	2	2
ST056186	0	0	0	0	0	0	0	0	0	0	0	0	0	0	0	0	0	0	0	0	0	0	0	0	0	0	0	0	0
ST056188	0	0	0	0	0	0	0	0	0	0	0	0	0	0	0	0	0	0	0	0	0	0	0	0	0	0	0	0	0
ST056220	1	1	1	1	1	1	1	1	1	1	1	1	1	1	1	1	1	2	1	1	1	1	1	1	1	1	1	1	1
ST056283	1	1	1	1	1	1	1	1	1	1	1	1	1	1	1	1	1	1	1	1	1	1	1	1	1	1	1	1	1
ST056285	1	1	1	1	1	1	1	1	1	1	1	1	1	1	1	1	1	1	1	5	1	1	1	1	1	1	1	1	1
ST056287	0	0	0	0	0	0	0	0	0	0	0	0	0	0	0	0	0	0	0	0	0	0	0	0	0	0	0	0	0
ST056289	1	1	1	1	1	1	1	1	1	1	1	1	1	1	1	1	1	1	1	1	1	1	1	1	1	1	1	1	1
ST056292	4	4	4	5	4	5	4	5	5	5	1	4	4	4	5	4	5	5	5	5	5	5	5	4	5	4	4	5	4
ST056301	1	1	1	1	1	1	1	1	1	1	1	1	1	1	1	1	1	1	1	1	1	1	1	1	1	1	1	1	1
ST056305	1	1	1	1	1	1	1	1	1	1	1	1	1	1	1	1	1	1	1	1	1	1	1	1	1	0	0	1	1
ST056306	2	2	2	3	2	3	2	3	3	3	2	2	2	2	3	2	3	3	3	3	3	3	3	2	2	2	2	2	2
ST056307	0	0	0	0	0	0	0	0	0	0	0	0	0	0	0	0	0	0	0	0	0	0	0	0	0	0	0	0	0
ST056311	0	0	0	0	0	0	0	0	0	0	1	0	0	0	0	0	0	2	0	1	0	0	0	0	0	0	0	0	0
ST056310	1	1	1	1	1	1	1	1	1	1	1	1	1	1	1	1	2	2	2	2	2	2	1	1	1	1	1	1	1
ST056312	0	0	0	0	0	0	0	0	0	0	0	0	0	0	0	0	0	0	0	0	0	0	0	0	0	0	0	0	0
ST056341	0	0	0	1	0	0	1	1	0	1	1	0	1	1	1	0	1	1	1	1	1	1	1	1	0	0	0	1	1

ST056348	0	0	0	0	0	0	0	1	0	0	1	0	0	0	0	0	1	1	1	1	1	1	0	0	0	0	0	0	0
ST056353	4	3	4	3	3	3	3	3	3	3	3	4	3	4	4	3	4	4	4	4	4	3	3	3	3	3	3	3	3
ST056390	4	4	4	4	4	4	4	4	4	4	4	4	4	4	4	4	4	4	4	4	4	4	4	4	4	4	4	4	4
ST056919	0	0	0	0	0	0	0	0	0	0	0	0	0	0	0	0	0	0	0	0	0	0	0	0	0	0	0	0	0
ST056939	0	0	0	0	0	0	0	0	0	0	0	0	0	0	0	0	0	0	0	0	0	0	0	0	0	0	0	0	0
ST056941	0	0	0	0	0	0	0	0	0	0	0	0	0	0	0	0	0	0	0	0	0	0	0	0	0	0	0	0	0
ST057073	1	1	1	1	1	1	1	1	1	1	1	1	1	1	1	1	1	1	1	1	1	1	1	1	1	0	0	1	1
ST057075	0	0	0	0	0	0	0	0	0	0	0	0	0	0	0	0	0	0	0	0	0	0	0	0	0	0	0	0	0
ST057076	0	0	0	0	0	0	0	0	0	0	0	0	0	0	0	0	0	0	0	0	0	0	0	0	0	0	0	0	0
ST057091	0	0	0	0	0	0	0	0	0	0	0	0	0	2	0	0	0	3	3	4	2	0	0	0	0	0	0	0	2
ST057093	0	0	0	0	0	0	0	0	0	0	0	0	0	0	0	0	0	0	0	0	0	0	0	0	0	0	0	0	0
ST057095	0	0	0	0	0	0	0	0	0	0	0	0	0	0	0	0	0	0	0	0	0	0	0	0	0	0	0	0	0
ST057098	0	0	0	0	0	0	0	0	0	0	0	0	0	0	0	0	0	0	0	0	0	0	0	0	0	0	0	0	0
ST057163	0	0	0	0	0	0	0	0	0	0	0	0	0	0	0	0	0	0	0	0	0	0	0	0	0	0	0	0	0
ST057165	1	1	1	1	1	1	1	1	1	1	1	1	1	1	1	1	1	1	1	1	1	1	1	1	1	1	0	1	1
ST057172	1	1	1	1	1	1	1	1	1	1	1	1	1	1	1	1	1	2	1	1	1	1	1	1	1	1	1	1	1
ST057175	0	0	0	0	0	0	0	0	0	0	0	0	0	0	0	0	0	0	0	0	0	0	0	0	0	0	0	0	0
ST057182	0	0	0	0	0	0	0	0	0	0	0	0	0	0	0	0	0	0	0	0	0	0	0	0	0	0	0	0	0
ST057212	1	1	1	1	1	1	1	1	2	1	1	1	1	1	1	1	2	2	2	2	2	2	1	1	1	1	1	1	1
ST057225	4	4	4	4	4	4	4	4	4	4	4	4	4	4	4	4	4	4	4	4	4	4	4	4	4	4	4	4	4
ST057232	0	0	0	0	0	0	0	1	0	0	1	0	0	0	0	0	1	1	1	1	1	1	0	0	0	0	0	0	0
ST057233	1	1	1	1	1	1	1	1	1	1	1	1	1	1	1	1	1	1	1	1	1	1	1	1	1	1	1	1	1

ST057240	0	0	0	0	0	0	0	0	0	0	0	0	0	0	0	0	0	0	0	0	0	0	0	0	0	0	0	0	0
ST057251	1	1	2	2	1	2	2	2	2	2	2	2	1	1	2	1	2	2	2	2	2	2	2	1	2	1	1	1	2
ST057253	0	0	0	0	0	0	0	0	0	0	0	0	0	0	0	0	0	0	0	0	0	0	0	0	0	0	0	0	0
ST057257	0	0	0	0	0	0	0	0	0	0	0	0	0	0	0	0	0	0	0	0	0	0	0	0	0	0	0	0	0
ST057259	0	0	0	0	0	0	0	0	0	0	0	0	0	0	0	0	0	0	0	0	0	0	0	0	0	0	0	0	0
ST057260	0	0	0	0	0	0	0	0	0	0	0	0	0	0	0	0	0	0	0	0	0	0	0	0	0	0	0	0	0
ST057261	0	0	0	1	0	0	1	1	0	1	1	1	1	1	1	0	1	1	1	1	1	1	0	1	0	0	0	1	1
ST057529	0	0	0	0	0	0	0	0	0	0	0	0	0	0	0	0	0	0	0	0	0	0	0	0	0	0	0	0	0
ST057531	2	2	2	2	2	2	2	2	2	2	2	2	2	2	2	2	2	2	2	2	2	2	2	2	2	2	2	2	2
ST057580	1	1	1	1	1	1	1	1	1	1	1	1	1	1	1	1	2	2	2	2	2	2	1	1	1	1	1	1	1
ST057602	1	1	1	1	1	1	1	2	2	2	1	1	1	1	1	1	2	2	2	2	2	2	2	1	2	1	1	1	1
ST057705	1	1	1	1	1	1	1	1	1	1	1	1	1	1	1	1	1	1	1	1	1	1	1	1	1	0	0	1	1
ST057771	0	0	0	0	0	0	0	0	0	0	0	0	0	0	0	0	0	0	0	0	0	0	0	0	0	0	0	0	0
ST059837	1	1	1	1	1	1	1	1	1	1	1	1	1	1	1	1	1	1	1	1	1	1	1	1	1	1	1	1	1
ST066914	3	3	3	3	3	3	3	3	3	3	3	3	3	3	3	3	3	3	3	3	3	3	3	3	3	3	3	3	3
ST069307	1	1	1	1	1	1	1	1	1	1	1	1	1	1	1	1	1	2	2	2	2	2	1	1	1	1	1	1	1
ST069312	1	1	1	1	1	1	1	1	1	1	1	1	1	1	1	1	1	1	1	1	1	1	1	1	1	1	1	1	1
ST069321	2	2	2	3	2	2	2	3	3	3	2	2	2	2	2	2	3	3	3	3	3	3	2	2	2	2	2	2	2
ST069324	1	1	1	1	1	1	1	1	1	1	1	1	1	1	1	1	1	1	1	1	1	1	1	1	1	1	1	1	1
ST069327	0	0	0	0	0	0	0	0	0	0	0	0	0	0	0	0	0	0	0	0	0	0	0	0	0	0	0	0	0
ST069329	1	1	1	1	1	1	1	1	1	1	1	1	1	1	1	1	1	1	1	1	1	1	1	1	1	1	1	1	1
ST069334	4	4	4	4	4	4	4	4	4	4	4	4	4	4	4	4	4	4	4	4	4	4	4	4	4	4	4	4	4

ST069335	0	0	1	1	0	0	1	1	1	1	1	0	1	1	1	0	1	2	1	1	1	1	0	1	1	0	0	1	1
ST069355	2	2	2	2	1	2	2	2	2	2	2	2	2	2	2	1	2	2	2	2	2	2	2	2	2	1	1	2	2
ST069364	0	0	0	0	0	0	0	0	0	0	0	0	0	0	0	0	0	0	0	0	0	0	0	0	0	0	0	0	0
ST069381	0	0	1	1	0	0	1	1	1	1	1	0	1	1	1	0	1	1	1	1	1	1	1	1	0	0	0	1	1
ST072163	0	0	0	0	0	0	0	0	0	0	0	0	0	0	0	0	0	0	0	0	0	0	0	0	0	0	0	0	0
ST072172	0	0	0	0	0	0	0	0	0	0	0	0	0	0	0	0	0	0	0	0	0	0	0	0	0	0	0	0	0
ST072187	5	4	5	4	5	4	4	5	5	5	4	4	4	4	4	4	4	4	4	5	5	4	4	4	5	4	4	5	4
ST073359	0	0	0	0	0	0	0	0	0	0	0	0	0	0	0	0	0	0	0	0	0	0	0	0	0	0	0	0	0
ST075197	4	4	4	4	4	4	4	4	4	4	4	4	4	4	4	4	4	4	4	4	4	4	4	4	4	4	4	4	4
ST075201	4	4	4	4	4	4	4	4	4	4	4	4	4	4	4	4	4	4	4	4	4	4	4	4	4	4	4	4	4
ST079379	0	0	0	0	0	0	0	0	0	0	0	0	0	0	0	0	0	0	0	0	0	0	0	0	0	0	0	0	0
ST085767	5	3	5	3	3	3	2	3	3	3	4	3	4	4	2	2	4	3	5	5	5	3	3	3	2	2	2	3	2
ST095786	0	0	0	1	0	0	0	1	0	1	1	0	1	1	1	0	1	1	1	1	1	1	0	0	0	0	0	0	1
ST098734	1	0	1	1	0	1	1	1	1	1	1	1	1	1	1	0	1	1	1	1	1	1	1	1	1	0	0	1	1
ST098733	4	5	5	4	5	5	5	5	5	5	5	5	5	5	5	5	5	5	5	5	5	5	5	5	5	5	5	5	4
ST057235	1	1	1	1	1	1	1	1	1	1	1	1	1	1	1	1	1	1	1	1	1	1	1	1	1	1	1	1	1
ST065835	2	2	2	2	2	2	2	2	2	2	2	2	2	2	2	2	2	2	2	3	2	2	2	2	2	2	2	2	2
ST047334	0	0	1	1	0	0	1	1	1	1	1	0	1	1	1	0	1	1	1	1	1	1	1	1	1	0	0	1	1
ST092303	0	0	0	0	0	0	0	0	0	0	0	0	0	0	0	0	0	0	0	0	0	0	0	0	0	0	0	0	0
ST095415	0	0	0	0	0	0	0	0	0	0	0	0	0	0	0	0	0	0	0	0	0	0	0	0	0	0	0	0	0
ST096004	0	0	0	0	0	0	0	0	0	0	1	0	0	0	0	0	0	0	1	1	0	1	0	0	0	0	0	0	0
ST097774	0	0	1	1	0	0	1	1	0	1	1	0	1	1	1	0	1	1	1	1	1	1	0	1	0	0	0	1	1

ST056352	1	1	1	1	1	1	1	1	1	1	1	1	1	1	1	1	1	2	2	2	2	2	1	1	1	1	1	1	1

Table S6 Predictions of antiproliferative activity of select NPL-720 compounds (the 106 with available experimental data) against the remaining 29 cell lines the network was trained on.
